# Tick genomics through a Nanopore: a low-cost approach for tick genomics

**DOI:** 10.1186/s12864-025-11733-4

**Published:** 2025-07-01

**Authors:** Christina Meiring, Monique Eygelaar, Josephus Fourie, Michel Labuschagne

**Affiliations:** 1 Clinglobal, B03/04, The Tamarin Commercial Hub, Tamarin, 90903 Mauritius; 2 Clinomics, Uitzich Road, Bainsvlei, Bloemfontein, 9338 South Africa; 3 Clinvet International Pty (Ltd), 1479 Talmadge Hill South, Waverly, NY 14892 USA

**Keywords:** Genome assembly, Long reads, Nanopore, Cloud computing, tick genome, De novo

## Abstract

**Background:**

The assembly of large and complex genomes can be costly since it typically requires the utilization of multiple sequencing technologies and access to high-performance computing, while creating a dependency on external service providers. The aim of this study was to independently generate draft genomes for the cattle ticks *Rhipicephalus microplus* and *R. appendiculatus* using Oxford Nanopore sequencing technology.

**Results:**

Exclusively, Oxford Nanopore sequence data were assembled with Shasta and finalized on the Amazon Web Services cloud platform, capitalizing on the availability of up to 90% discounted Spot instances. The assembled and polished *R. microplus* and *R. appendiculatus* genomes from our study were comparable to published tick genomes where multiple sequencing technologies and costly bioinformatic resources were utilized that are not readily accessible to low-resource environments. We predicted 52,412 genes for *R. appendiculatus*, with 31,747 of them being functionally annotated. The *R. microplus* annotation consisted of 60,935 predicted genes, with 32,263 being functionally annotated in the final file. The sequence data were also used to assemble and annotate genetically distinct *Coxiella*-like endosymbiont genomes for each tick species. The results indicated that each of the endosymbionts exhibited genome reductions. The Nanopore Q20 + library kit and flow cell were used to sequence the > 80% AT-rich mitochondrial DNA of both tick species. The sequencing generated accurate mitochondrial genomes, encountering imperfect base calling only in homopolymer regions exceeding 10 bases.

**Conclusion:**

This study presents an alternative approach for smaller laboratories with limited budgets to enter the field and participate in genomics without capital intensive investments, allowing for capacity building in a field normally exclusively accessible through collaboration and large funding opportunities.

**Supplementary Information:**

The online version contains supplementary material available at 10.1186/s12864-025-11733-4.

## Background

Participation in the exploration of genomes and the generation of meaningful genomic data requires access to a service provider to generate sequence data, and a bioinformatics connection for processing the generated data in a fit for purpose format [1]. Working with a complex genome with significant repeat content limits the options of service providers for both sequence generation and processing, especially within an African context.

Cost-effective genomics will allow small laboratories with limited budgets to participate and contribute toward the current understanding of biology (from an individual laboratory perspective). Short read sequence technologies paved the way for the *de novo* assembly of smaller genomes with high accuracy and low cost per giga base (Gb) [2]. However, due to their relatively short read lengths of < 400 bases, it resulted in highly fragmented *de novo* assemblies of complex genomes where repeat regions exceeded the maximum short read length [3]. This necessitated the development of long-read sequencing technologies to span the repeat regions and allow the generation of more accurate *de novo* genome assemblies [4]. Nanopore sequencing (Oxford Nanopore Technologies; ONT) is one such technology in which the sequence read length is dependent on the length of the prepared library, effectively enabling reads with an excess of 2 million bases (2.3 Mb) [5]. Nanopore technology also allows for the inhouse establishment of the technology at a relatively low cost [6], since a starter pack including the sequencing device, four flow cells, and a sequencing kit can be purchased for USD 3,250 when the study was initiated in the last quarter of 2021. This bundle provides all the necessary components to transition from DNA to sequence data and utilizes reagents and consumables that are available in most molecular laboratories.

Advancements in high-throughput sequencing technologies, coupled with the reduced cost of sequencing, have led to exponential growth of more complex biological sequence data [7]. In addition to generating raw sequence data, storage and analyses of such large datasets can be challenging. This can create a gap between the amount of data being generated and the available computational resources of users. Researchers therefore often require high-performance computing (HPC) infrastructures, with a large amount of available storage [8, 9]. These infrastructures require a significant capital investment and can become expensive and challenging to set up and maintain. It is therefore not a feasible option for individuals aiming to conduct genomic studies using the available technology. Over the years, cloud computing has become a promising solution to address these challenges. Cloud computing offers users a “pay-as-you-go” option where you only pay for the resources you are using when you are using it [9]. Researchers are therefore no longer limited by their available “on-site” computational capacities and now have access to “limitless” resources and storage, at a reasonable cost, permitted that you have access to a stable broadband internet connection. Amazon Web Services (AWS) offer users the option to take advantage of unused resources available in the AWS cloud (known as “Spot Instances”) at a discount of up to 90% (Amazon EC2 Spot Instances).

Ticks have large and complex genomes spanning over 2 giga base pairs (Gbp), with an estimated repeat content of between 52.6 and 64.4% [10]. Consequently, generating high quality reference genomes for tick species has been a great challenge. Tick genome sequencing and assembly endeavours have been undertaken utilizing multiple sequencing platforms to resolve the assembly of repeats and to maintain or increase accuracy and throughput [10–13]. However, the costs and resources necessary to accomplish this reduce the reproducibility of this workflow. Since the costs associated with nanopore sequencing (instruments and consumables) are lower than those associated with other sequencing platforms [6, 14], this study aimed to use long-read nanopore sequencing technology to produce draft genome assemblies for two tick species, namely, *R. microplus* and *R. appendiculatus.* The sequence data were also used to assemble the mitochondrial genomes for each tick species. We envisage that using a single long-read sequencing technology to produce assemblies of these ticks will be comparable to genomes that were generated using hybrid sequencing. The availability of affordable technology for the generation of high-quality genomes provides opportunities for increased participation in genome projects.

Ticks are known to carry a wide variety of pathogens, affecting both animal and human health globally and contributing significantly to economic losses [15]. *Rhipicephalus microplus* is a major vector of pathogens, such as *Babesia bovis*, which causes bovine babesiosis, a disease responsible for decreased milk and beef production [16]. Consequently, *R. microplus* has been extensively studied, particularly in the context of acaricide resistance, genomics, and its role in pathogen transmission [10, 17, 18]. In contrast, *R. appendiculatus*, although less well-studied, poses a significant threat as the primary vector of *Theileria parva*, the causative agent of East Coast fever in cattle. This disease is one of the most devastating tick-borne infections in sub-Saharan Africa, with mortality rates exceeding 40% in infected herds and severe economic implications for livestock-dependent communities [19, 20]. Ticks not only carry pathogens but also harbor a diversity of commensal and symbiotic organisms. Among these, symbiotic bacteria have become well studied and are known to play essential roles in host fitness, nutritional adaptation, development, and reproduction [21, 22]. One of the most abundant endosymbionts found in ticks is the maternally inherited *Coxiella*-like endosymbiont (CLE) bacteria, which is closely related to the Q-fever causing *Coxiella burnetii*. This study also aimed to identify these bacterial genomic segments within the sequencing data derived from the DNA isolated from the two tick species, with the objective of generating bacterial assemblies.

The generation of draft genome assemblies of the cattle ticks *R. microplus* and *R. appendiculatus* is presented here, employing solely MinION-based nanopore technology for sequence data generation. High molecular weight (HMW) DNA was isolated using instruments and procedures readily available in a standard molecular laboratory. Subsequently, these repeat-rich genomes were sequenced using a single sequencing technology, and the analysis of the base called DNA was conducted on a cloud-based computing platform. The analysis of the assembled genomes revealed that the generated data were comparable with available genomes for *R. microplus* from two independent sources [10, 12]. We subsequently used the same approach to generate the first draft nuclear genome for *R. appendiculatus* and demonstrated that the generated AT-rich mitochondrial genome could be *de novo* assembled with high accuracy using Q20 + Nanopore sequencing chemistry. The results also provide insight into the unique endosymbionts present in these laboratory-raised tick isolates.

## Results

### Genomic DNA isolation, quality control, and sequencing

**Fig. 1:**
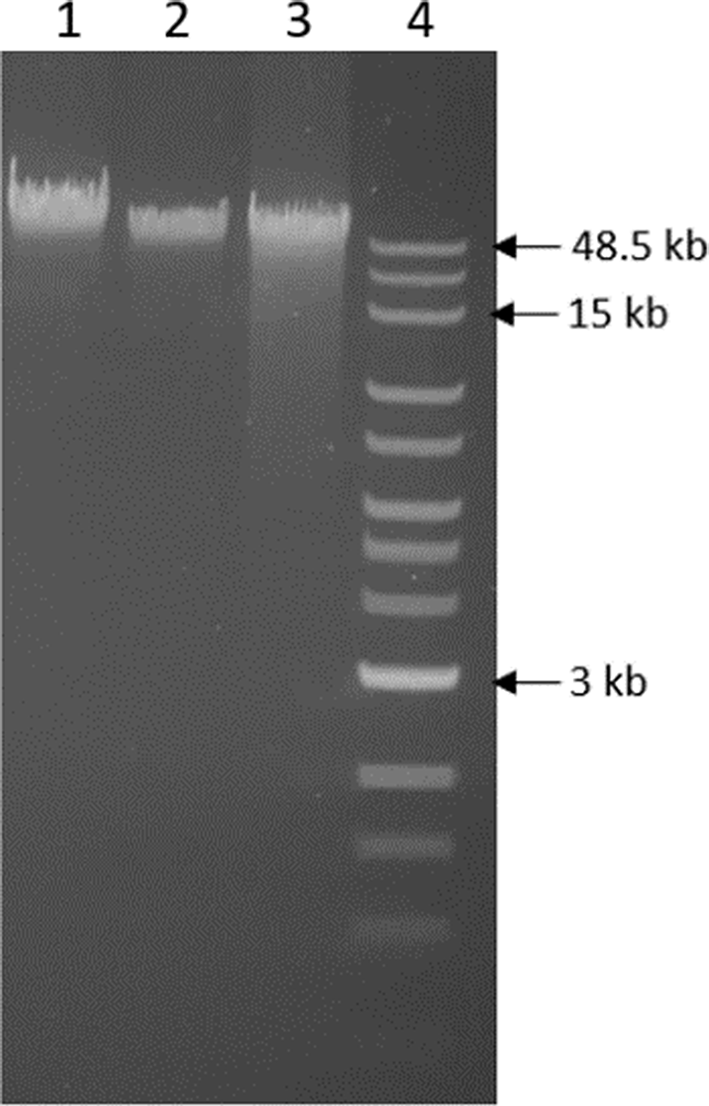
Agarose gel electrophoresis as a tool for quality control of isolated tick DNA. *R. microplus* gDNA (225 ng; lane 1), *R. appendiculatus* gDNA (115 ng; lane 2) and Lambda DNA (250 ng; lane 3). The NEB 1 kb extended ladder was loaded in lane 4

Phenol-extracted and ethanol-precipitated gDNA was subjected to agarose gel electrophoresis as a simple quality control step to evaluate the isolated DNA. Electrophoretic mobility of the isolated DNA was compared to the NEB 1 kb extended DNA ladder and indicated that the isolated DNA was of high molecular weight (≥ 48 kbp) with minimal degradation (Fig. 1). The total DNA yield from approximately 1.25 g *R. microplus* and *R. appendiculatus* eggs as starting material was approximately 1 mg.

The HMW DNA was subjected to an initial round of library preparation, resulting in clumping of the magnetic beads during the initial attempt and subsequent failure to recover enough DNA for library preparation (data not shown). Subsequently, DNA was sheared using a 29G needle and a Nest P1000 pipette tip, and the sheared DNA was used as a template in the ONT SQK-LSK109 kit for library preparation. The MinION Mk1B device was connected to a laptop with a solid-state drive using a USB3 cable for data collection. Shearing resulted in noticeable differences in yield and read length from a single flow cell run (Table 1). This increase in read length resulted in a clear drop in yield from a single flow cell with the P1000 sheared DNA compared to the 29G sheared DNA.

**Table 1:** Impact of *R. microplus* genomic DNA shearing on ONT SQK-LSK109 library construction and flow cell output (results are averages obtained from triplicate runs)

	P1000	29G
Mean read length (bases):	16,213	10,579
Mean read quality (Q-score):	12	12
Number of reads:	143,296	763,671
Read length N50:	44,342	13,142
STDEV read length:	22,685	6,061
Total bases:	2,308,668,051	8,073,105,391

The P1000 sheared DNA allowed for the generation of long reads to aid in the assembly, while the 29G sheared DNA generated higher yields, resulting in better coverage of the target genomes per unit of flow cell used. The yield per flow cell was also increased by introducing more frequent DNase I treatment and reloading of the flow cell at 12-hour intervals. This approach allows for multiple loading of a single library on a flow cell and increases the yield from a single flow cell by degrading and releasing the entangled DNA occupying functional pores and making the pore available for sequencing during a subsequent reloading of the flow cell [23]. Sequential washing and reloading resulted in the generation of sufficient sequence data of different lengths using 10 flow cells per final assembled genome.

### Base calling of sequence data

A local computer with an NVIDIA GeForce RTX 2080 Ti graphics card was used for GPU base calling, and the resulting FASTQ files, which are approximately 8 times smaller in size than the fast5 files, allowing faster uploads to AWS in a bandwidth limited setting.

Reads were initially base called using the “high accuracy” (HAC) base calling model (95% single read accuracy), which at the time was the most accurate base calling model available. The reads were subjected to the “Super High Accuracy” (SUP) base calling model (98% single read accuracy) once it became available, allowing for a direct comparison between the base called datasets generated for the two models (Table 2).

**Table 2:** Base-called data generated for the *R. microplus* (RmCVSA) and *R. appendiculatus* (RaCVSA) sequence data using the HAC and SUP base-calling models (data generated using Q10 filtered reads as input)

	RmCVSA		RaCVSA	
	HAC	SUP	HAC	SUP
Mean read length	8,923	8,926	6,799	6,783
Mean read quality	14	15	14	15
Number of reads	14,697,828	15,425,088	20,859,835	22,411,932
Read length N50	14,470	14,626	10,018	9,887
Total bases	131,748,305,938	137,678,187,280	142,524,964,457	152,016,825,810

The results indicated that SUP base calling delivered superior data characteristics when compared with HAC after Q10 filtering, and it was decided to use both base-called datasets independently as input for the *de novo* genome assemblies.

### *De novo* tick genome assemblies

**Fig. 2:**
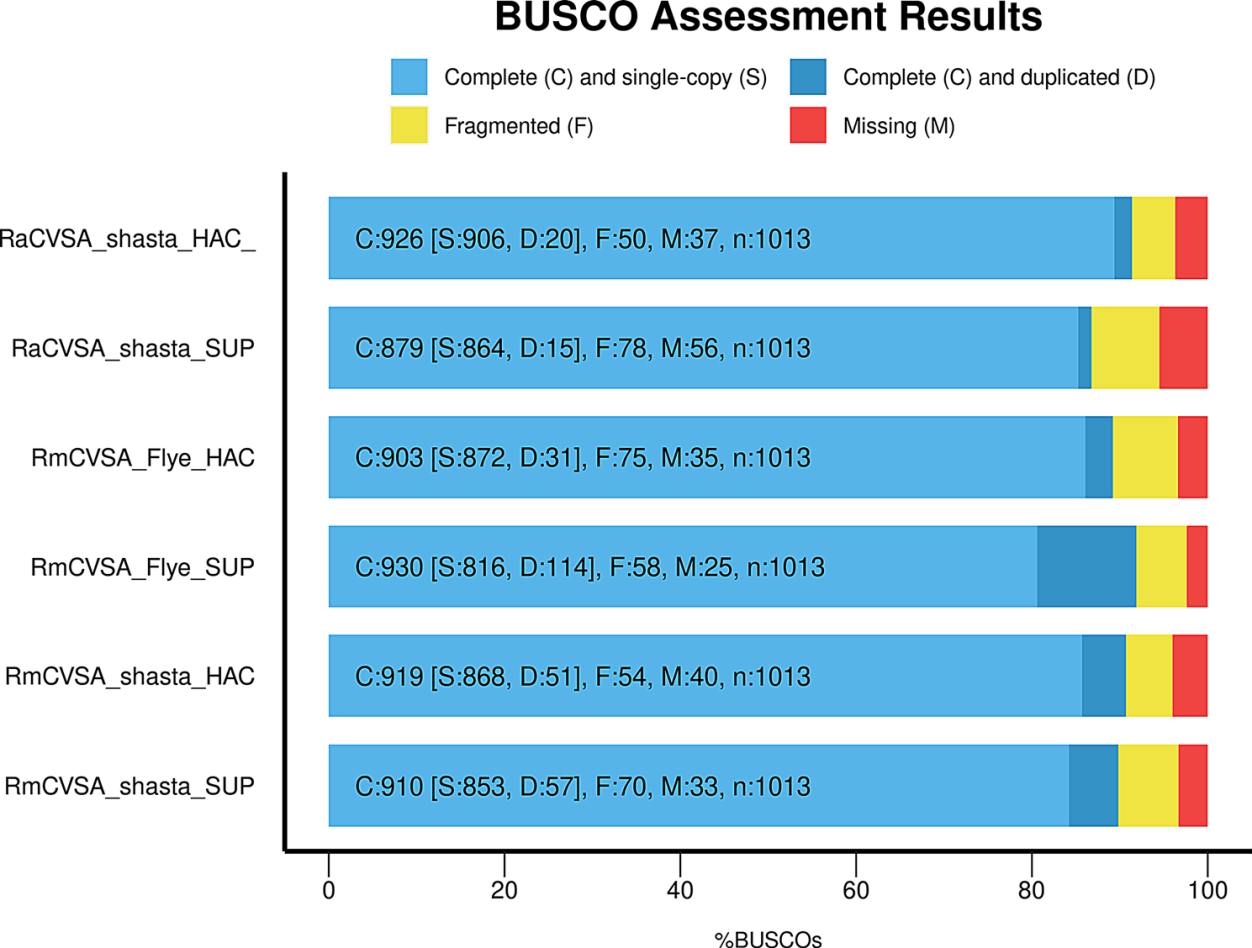
BUSCO analysis of the final tick assemblies making use of HAC and SUP base called data for RmCVSA and RaCVSA

Amazon Web Services Spot instances were used as computing resources to perform genome assembly and downstream processing of the assembled genomes. Shasta and Flye genome assemblers [24, 25] were used to generate genome assemblies for *R. microplus* using HAC and SUP base called data. Assembly results revealed that the Shasta assembled *R. microplus* genomes exhibited superior assembly statistics and proved to be more complete based on BUSCO scores when compared to the Flye results (Supplementary Table 1; Fig. 2). Shasta assemblies using HAC data resulted in assembly outputs superior to the SUP data assembly (Table 3; Supplementary Table 1). The final HAC RmCVSA nuclear genome assembly consisted of 11,359 contigs totalling 2.7 Gb, and the N50 contig length was 1.9 Mb, with the longest contig being 20.9 Mb (Table 3). The final RaCVSA assembly was 2.4 Gb in size, with 33,490 contigs, an N50 of 679.6 kb, and the longest contig was 20.8 Mb (Table 3). The GC content was 45.7% and 47% for RmCVSA and RaCVSA, respectively, which was similar to other tick species from a previous study, which also included *R. microplus* among the species [10].

**Table 3:** Summary of final assembly information for each tick species

Sample	Base calling	Assembled Bases	N50	Contigs	Longest Contig
RmCVSA (FINAL)	HAC	2,670,281,449	1,889,595	11,359	20,911,290
RmCVSA (FINAL)	SUP	2,678,591,779	1,707,119	11,544	26,072,894
RaCVSA (FINAL)	HAC	2,366,026,835	679,575	33,490	20,760,809
RaCVSA (FINAL)	SUP	2,545,458,806	686,602	23,030	19,469,694

A summary of the consumable and run-time costs of AWS instances used to run different software tools is presented in Table 4. The AWS instances are billed on one second increments. With the use of the Shasta assembler and AWS Spot instances, we assembled a genome for approximately USD 25 in only 5 h, compared to the Flye assembler, which ran consecutively for approximately six and a half days, costing USD 784.65 (Table 4).

**Table 4:** Typical capital investment, consumable and computing costs for sequencing and assembly of the RmCVSA tick genome in 2021

Activity	Item	Units needed	Unit cost (USD)	Flye (USD)	Shasta (USD)
**Capital investment**					
Sequencing	MinION device	1	1000.00	1000.00	1000.00
Base calling	GPU base calling PC	1	1700.00	1700.00	1700.00
**Total capital investment cost**:				**2700.00**	**2700.00**
**Consumable cost**					
Library prep and wash	Ligation sequencing kit and 3rd part materials	10	149.00	1490.00	1490.00
Sequencing	Flow cell	10	675.00	6750.00	6750.00
**Subtotal consumable cost**:				**8240.00**	**8240.00**
**AWS computing cost**		**Units needed (Flye/Shasta)**	**Unit cost (USD)**	**Total cost (Flye)**	**Total cost (Shasta)**
Assembly		155.63/5.0 h	5.0418	784.65	25.21
Polishing		64.35/144.0 h	0.6011	38.68	86.56
Purging		1/2.32 h	0.2765	0.28	0.64
Merging		NA/16 h	0.2765	NA	4.42
Storage space		1500 Gb	0.0174	26.10	26.10
**Subtotal cloud computing cost (USD)**:				**849.71**	**142.93**
**Total sequencing and assembly cost (USD)**:				**9089.71**	**8382.93**

The BUSCO analysis used to assess the completeness of each final Shasta assembly for the two tick species, using the HAC and SUP datasets, revealed that for both RmCVSA and RaCVSA, the final HAC assemblies had the best BUSCO scores (Fig. 2). The Shasta HAC-based assemblies contained the most complete single-copy orthologs, as well as the least number of missing single-copy orthologs compared to the SUP-based assemblies. The Flye2.9 RmCVSA assemblies exhibited marginally better BUSCO scores using the SUP dataset, but the assembly was still more incomplete than the Shasta RmCVSA HAC assembly. The Shasta assemblies, using the HAC base called data as input, resulted in superior assembly statistics (Supplementary Table 1) and BUSCO scores (Fig. 2), and were selected for further comparative analysis and submission to GenBank.

**Fig. 3:**
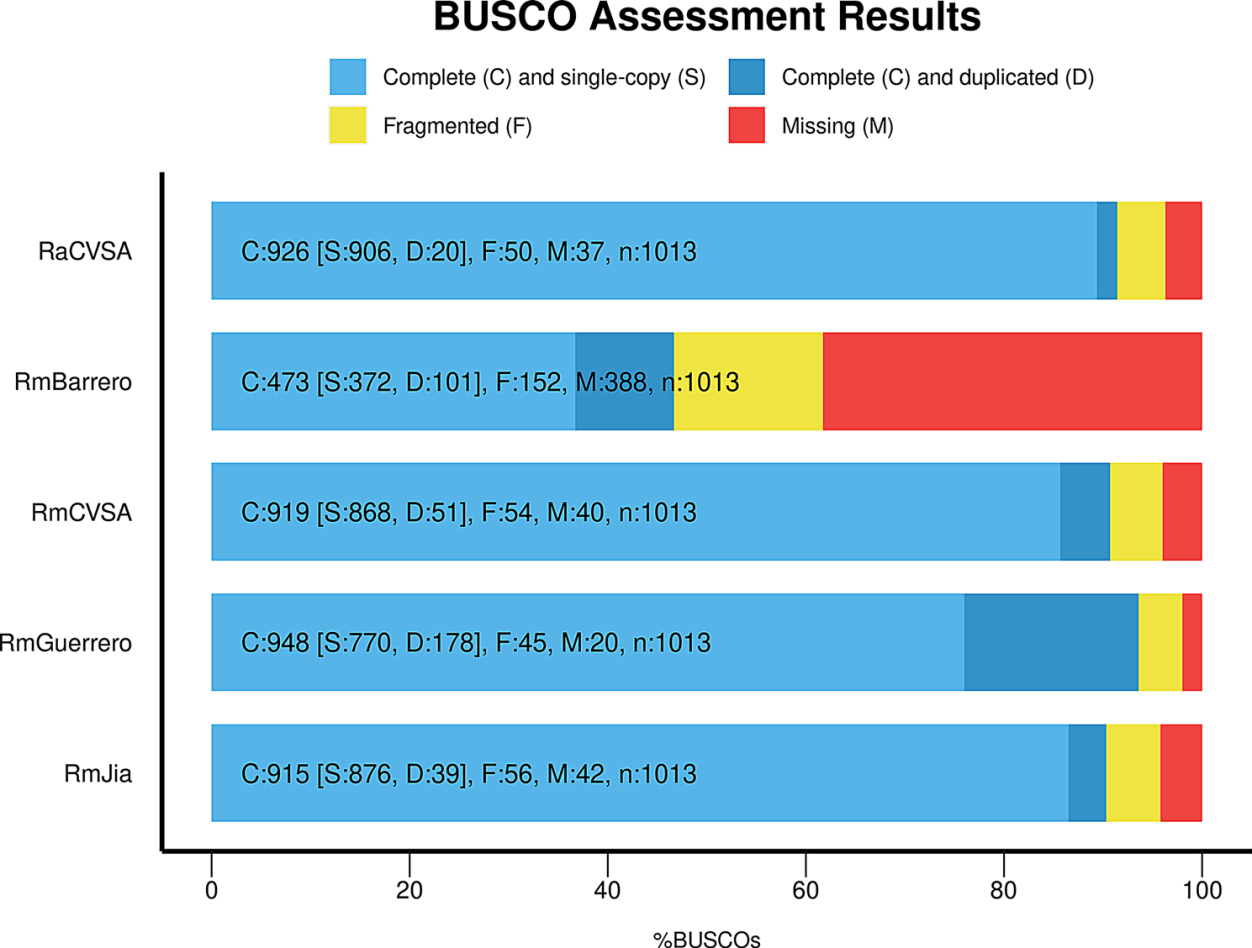
BUSCO analysis of *R. appendiculatus* (RaCVSA) and *R. microplus* (RmCVSA) genomes generated during this study as well as the *R. microplus* genomes available in GenBank

Comparative analysis of three *R. microplus* nuclear genomes (Table 5) based on assembly statistics (Table 6) and BUSCO analysis (Fig. 3) revealed that the current RmCVSA genome, based exclusively on Nanopore sequence data, yielded results comparable with other assemblies.

**Table 5:** Genome assembly reference data generated and used during this study

GenBank Accession	Identifier	Reference
PRJNA819877	RmCVSA	This study
PRJNA820528	RaCVSA	This study
CP094229	RmCVSA_CLE	This study
CP094378	RaCVSA_CLE	This study
OR545517	RmCVSA mtDNA	This study
OR545516	RaCVSA mtDNA	This study
ASM1343599v1	RmGuerrero	[12]
ASM1333972v1	RmJia	[10]
GCA_002176555.1	RmBarrero	[11]

**Table 6:** Assembly statistics of currently available *R. microplus* and *R. appendiculatus* genomes

	RmGuerrero	RmJia	RmBarrero	RmCVSA	RaCVSA
Assembled Bases	3,631,904,383	2,529,772,300	2,008,371,944	2,670,281,449	2,366,026,835
Contig N50	64,461	1,791,079	60,284	1,889,595	679,575
Contigs	93,737	8,624	280,135	11,359	33,490
Longest Contig	14,210,437	325,144,201	432,897	20,911,290	20,760,809
Genome Coverage	53 x	67 x	7 x	32 x	44 x

**Fig. 4:**
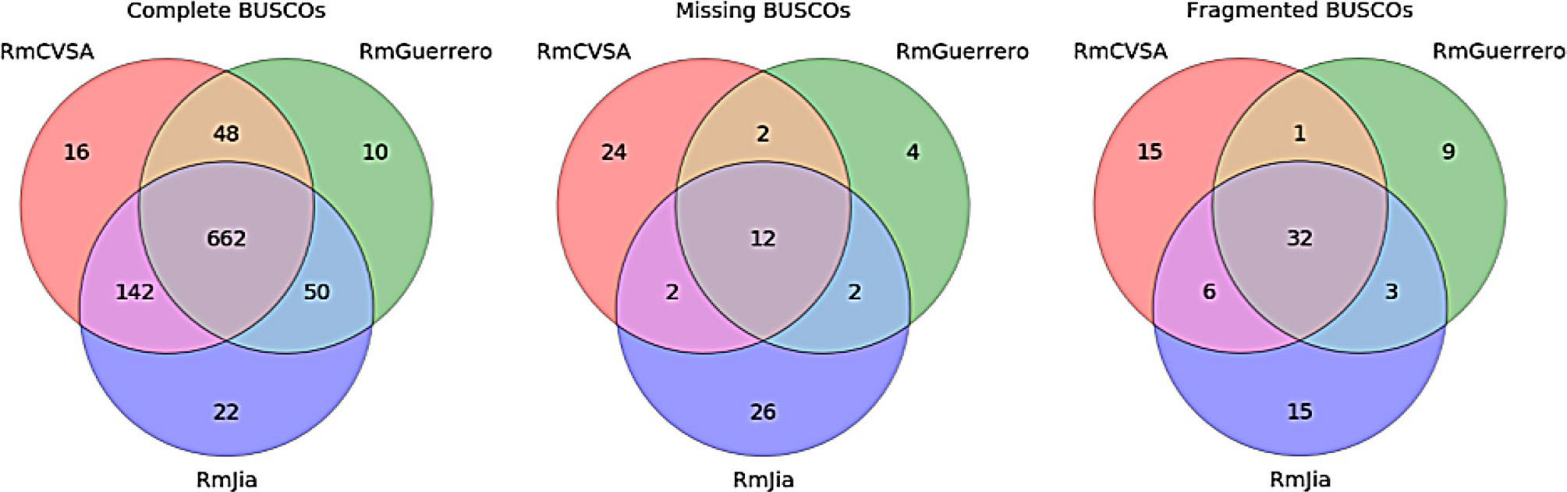
Venn diagram comparisons of the complete, missing, and fragmented BUSCO assessment results from the three available *R. microplus* genome assemblies

BUSCO scores (Fig. 3) obtained for each of the RmCVSA and RaCVSA genome assemblies indicated a high degree of genome completeness achieving a score of 90% for RmCVSA and 91% for RaCVSA, with 86% and 89% of genes being single-copy and complete, respectively (Fig. 3). Even higher scores were achieved when evaluating completeness using Compleasm (Supplementary Fig. 1), with RmCVSA containing 95% (90% single-copy) and RaCVSA containing 96% (95% single-copy) of the single copy orthologues present in the database. The BUSCO comparisons of the *R. microplus* genome assemblies revealed 662 common BUSCOs with the *R. microplus* genomes available on GenBank (Fig. 4). BUSCO data also revealed that the current work supplements the available genomic data for *R. microplus*, where 16 unique BUSCO targets were present in our assembly.

**Fig. 5:**
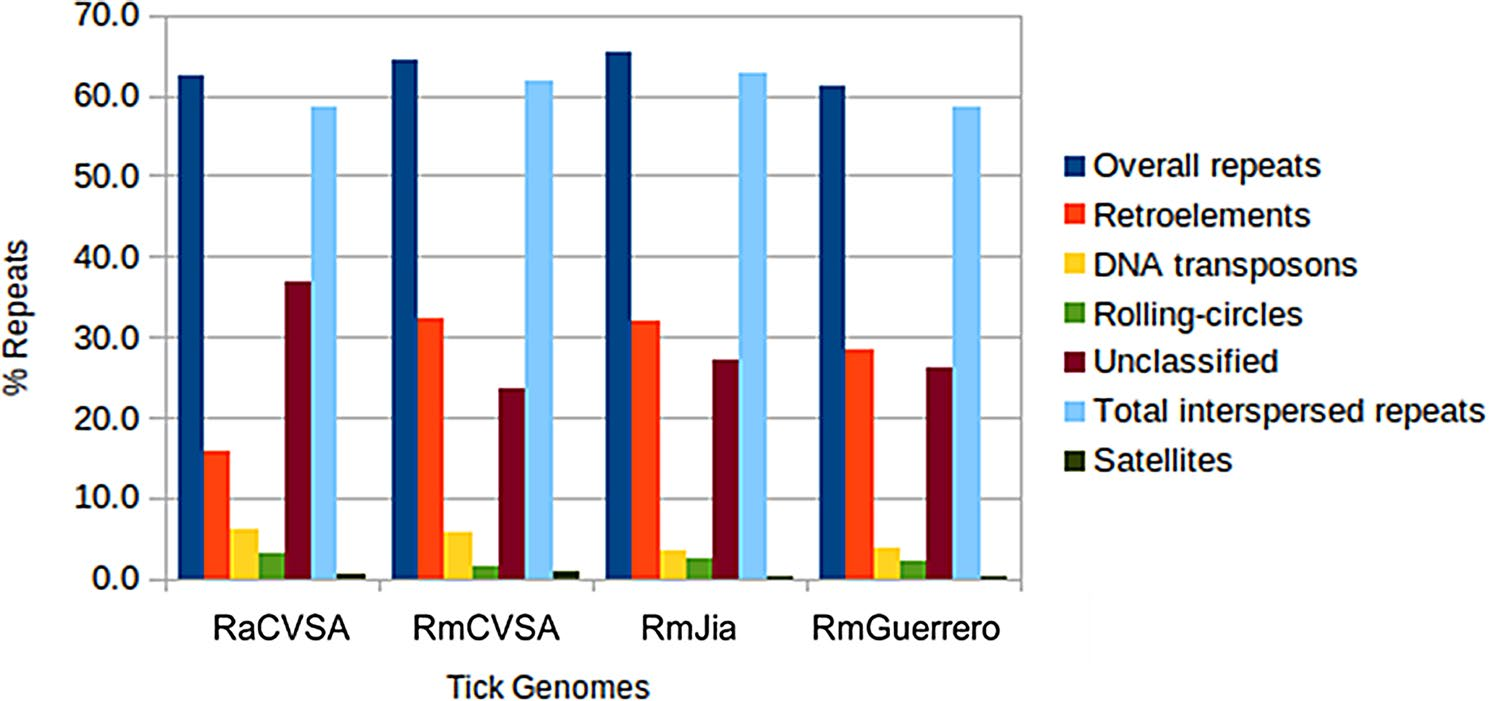
Repeat content of *R. appendiculatus* and *R. microplus* genomes from this study, and the *R. microplus* genomes available in GenBank

The overall repeat content between the two assembled genomes was 62.3% and 64.7% for RaCVSA and RmCVSA, respectively. This is comparable with what has previously been reported for a variety of different tick species [10]. For this study, we reassessed the repeat content for the available *R. microplus* genomes using the same version of *RepeatModele*r used to assess our genomes (Fig. 5). The most abundant repeat family identified in the genomes is interspersed repeats. For the *R. microplus* assemblies, the percentage of retroelements, which constitute mainly long interspersed nuclear elements (LINEs) and long terminal repeats (LTRs), nearly doubled compared to *R. appendiculatus*.

The repeat-masked genomes were passed to BRAKER3 for identification of gene models followed by EggNOG-mapper v2 and InterProScan for functional annotation. For RaCVSA (RaCVSA SR), BRAKER3 predicted 52,412 genes, of which 25,400 were supported by transcriptomic evidence, using short-read (SR) sialo transcript data and OrthoDB proteins (Arthropoda database) as input (Supplementary Table 2). For RmCVSA (RmCVSA SR), BRAKER3 identified 60,935 genes using SR sialo transcript data and OrthoDB proteins, with 30,501 of these genes supported by transcriptomic evidence.

The functional annotation of RaCVSA SR yielded a total of 31,747 genes annotated through EggNOG and InterProScan. Among these, 25,139 annotations were shared between the two tools (Supplementary Fig. 2). For the SR RmCVSA gene set, EggNOG and InterProScan functionally annotated a total of 32,263 genes. Within this set, 25,586 genes showed shared functional annotations across both tools (Supplementary Fig. 2).

Given the availability of an annotated genome for *R. microplus* (RmJia), the RmCVSA genome was also annotated using Liftoff, resulting in the transfer of 25,307 genes from RmJia to the RmCVSA assembly.

To evaluate the predicted proteins from the genome annotation, we used BUSCO in protein mode. The results indicated that the RaCVSA SR and RmCVSA SR annotations covered 90.70% and 89.30% of complete-copy genes from the Arthropoda gene set, respectively.

Following the submission of annotated genomes (RaCVSA SR and RmCVSA SR) to GenBank, RaCVSA SR retained a total of 52,408 genes, with 52,404 being protein-coding. For RmCVSA SR, the total gene count was 59,923, with 59,922 protein-coding genes (Supplementary Table 2). The BRAKER3 pipeline runtime for RaCVSA, after repeat masking of the genome assemblies, was approximately 5 days and 13 h using 14 cores of an Intel Core i9-9900 K CPU on a local machine. For RmCVSA, under similar conditions, the process took approximately 5 days and 22 h with the same configuration. The Liftoff annotation pipeline completed in approximately 15 min on a computer with 64 GB of RAM and an Intel Core i9-9900 K CPU, although only a single core was utilized.

### Mitochondrial genomes of two tick species

**Fig. 6:**
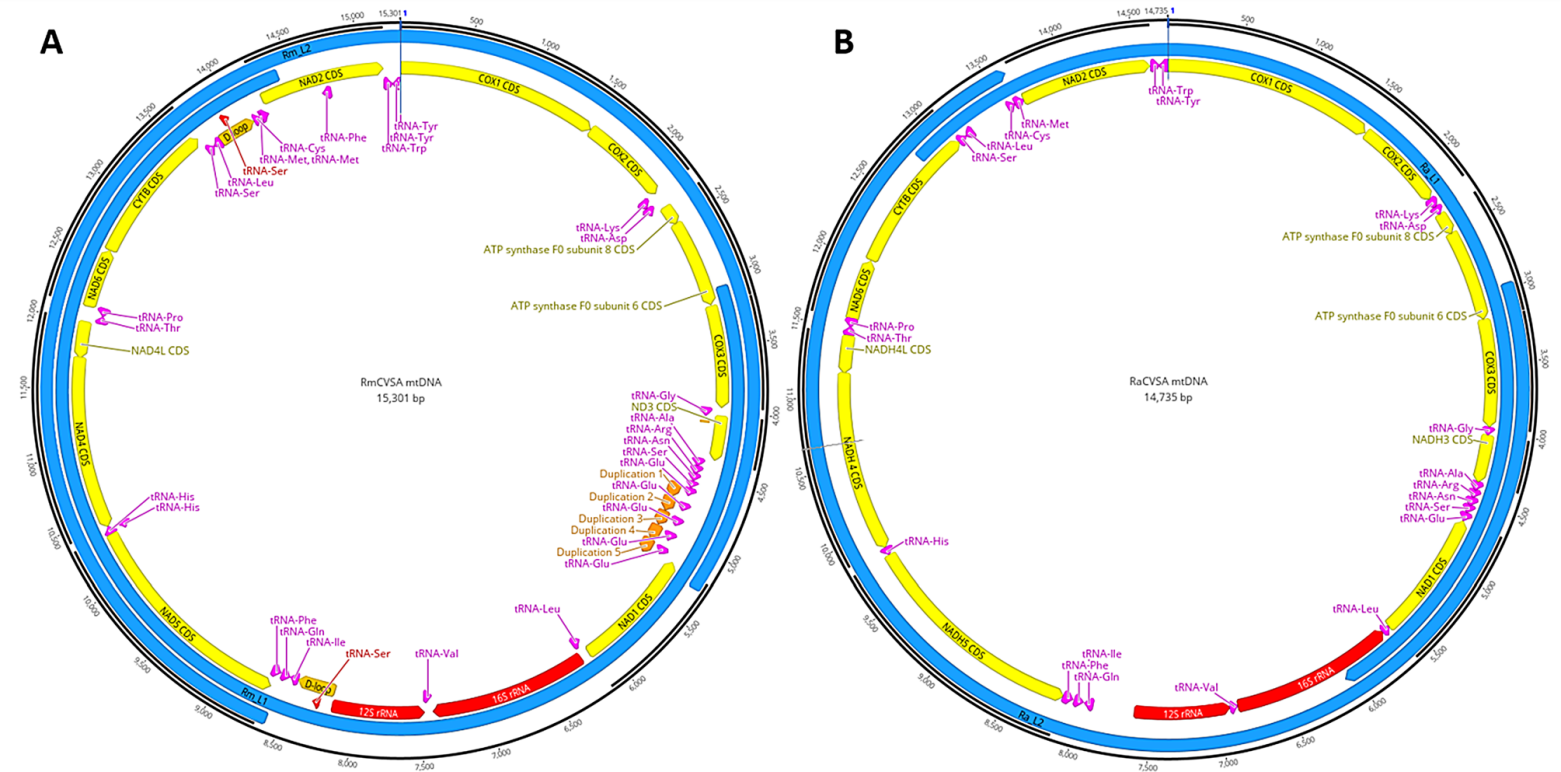
Mitochondrial DNA genome maps of the *R. microplus* CVSA (**A**) and *R. appendiculatus* CVSA (**B**) isolates. Long overlapping PCR target regions (annotated in light blue) are indicated as L1 and L2 and served as templates for the Q20 + sequencing workflow

The hard tick mtDNA genomes are AT rich (> 80%) with homopolymer stretches of ≥ 10 nucleotides dispersed throughout the mtDNA genome [26–28]. Tick mitochondrial contigs (based on ONT SQK-LSK109 and R9.4.1 flow cell-derived data) were identified and removed from the polished Shasta genome assemblies for both tick species. The extracted mitochondrial contigs were then *de novo* assembled using the Flye assembler. We obtained final mitochondrial assemblies in which the entire mitochondrial genome for each tick species was contained in a single contig. Pairwise comparisons with the reference mtDNA genomes revealed several discrepancies, mainly due to homopolymer stretches. Three rounds of Medaka polishing resulted in several errors still present in the assemblies, specifically homopolymer errors (data not shown). Q20 + sequencing of the overlapping long PCR products (Fig. 6A) resulted in a marked increase in the resolution of the homopolymer stretches, where only seven homopolymer stretches exceeding 10 homopolymer repeats, each missing a single nucleotide, were identified in the RmCSVA mtDNA genome. Sanger sequencing was used to confirm that the Q20 + data were missing a single homopolymer nucleotide in all seven repeats. A combination of Q20 + and Sanger data was used to generate to generate the final mtDNA genome sequence submitted to GenBank. The gene order and arrangement of the RmCVSA mtDNA were identical to those of available mtDNA genomes of *R. microplus* and contained 13 protein-coding genes, 2 rRNAs, and 26 tRNAs. The RmCVSA mtDNA genome (based on the genome generated from the genomic sequencing as well as the separate mtDNA genome generated from the overlapping long PCR amplicons) had five tandem repeat DNA sequences (with all 5 repeats exhibiting similar coverages on both genome DNA and PCR DNA derived reads) present, containing the 3’ end of NAD1 and Glu-tRNA.

The RaCVSA mtDNA genome (Fig. 6B) contained 13 protein-coding genes, 2 rRNAs, and 22 tRNAs and had identical gene order and arrangement to the *R. appendiculatus* mtDNA genomes available. RaCVSA mtDNA genome assembly based on Q20 + sequencing of both genomic and overlapping long PCR products resulted in a single 77.8% AT-rich contig with just a single T13 homopolymer stretch in the NADH 4 CDS being called T12, resulting in a frame shift. The homopolymer stretch was confirmed as T13 using Sanger sequencing and corrected in the final assembly. All other sequence variations generated using the Q20 + sequencing were confirmed using Sanger sequencing, and the final mtDNA genome sequence was submitted to GenBank.

**Fig. 7:**
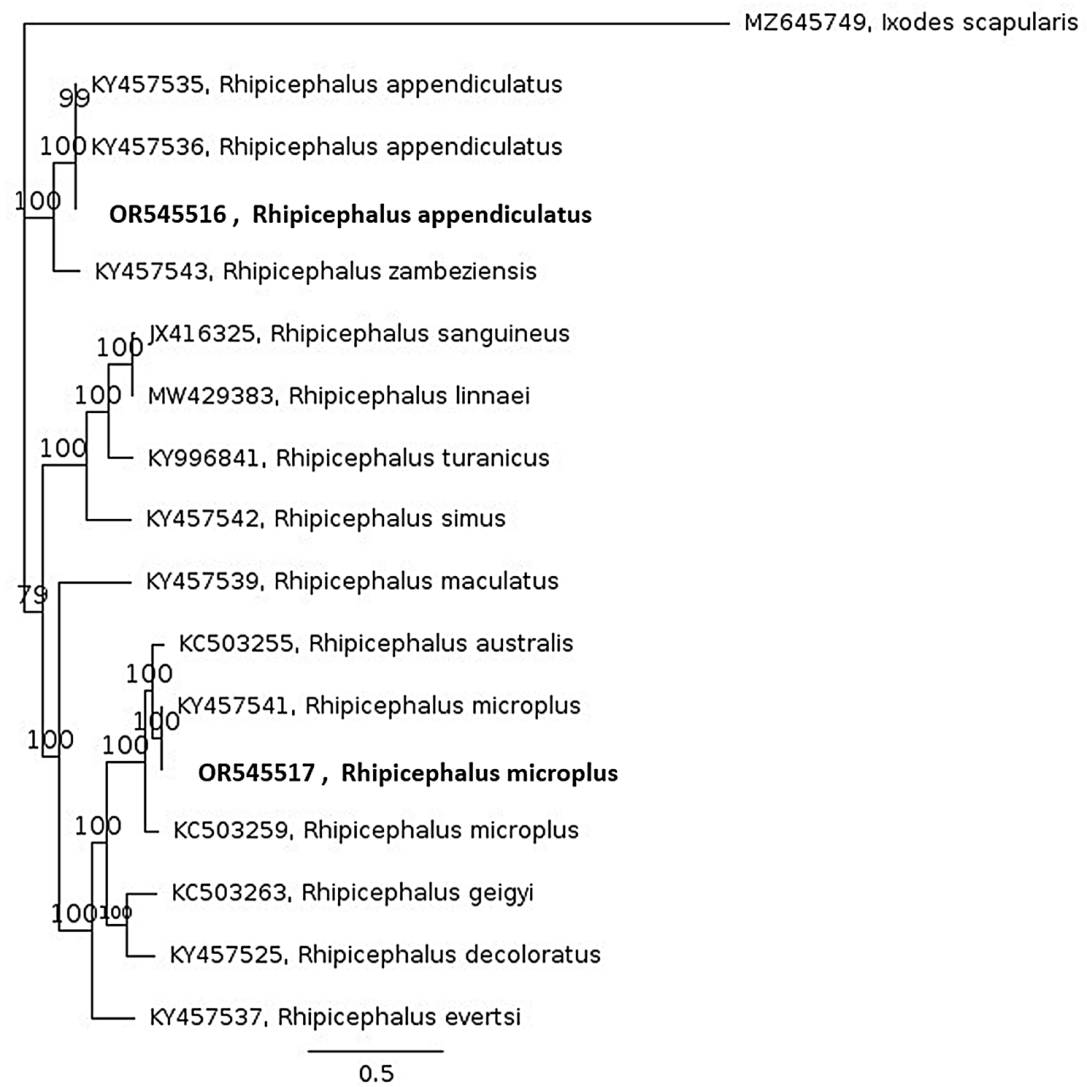
Bayesian inference phylogenetic analysis of *Rhipicephalus* spp. mitochondrial DNA sequences with the *Ixodes scapularis* mtDNA genome as the outgroup. The RmCVSA and RaCVSA isolates are highlighted in bold

The *R. microplus* and *R. appendiculatus* mtDNA genomes available in GenBank were subjected to multiple alignment and Bayesian inference phylogenetic analysis, and the RmCVSA and RaCVSA sequences were grouped within the *R. microplus* and *R. appendiculatus* clades, respectively (Fig. 7). Analysis of the mtDNA derived COX1 coding sequence revealed that the RmCVSA isolate were identical to isolates from Brazil (NC_023335) and Kenya (MT430985; MT430986).

### Bacterial genomes found in ticks

**Fig. 8:**
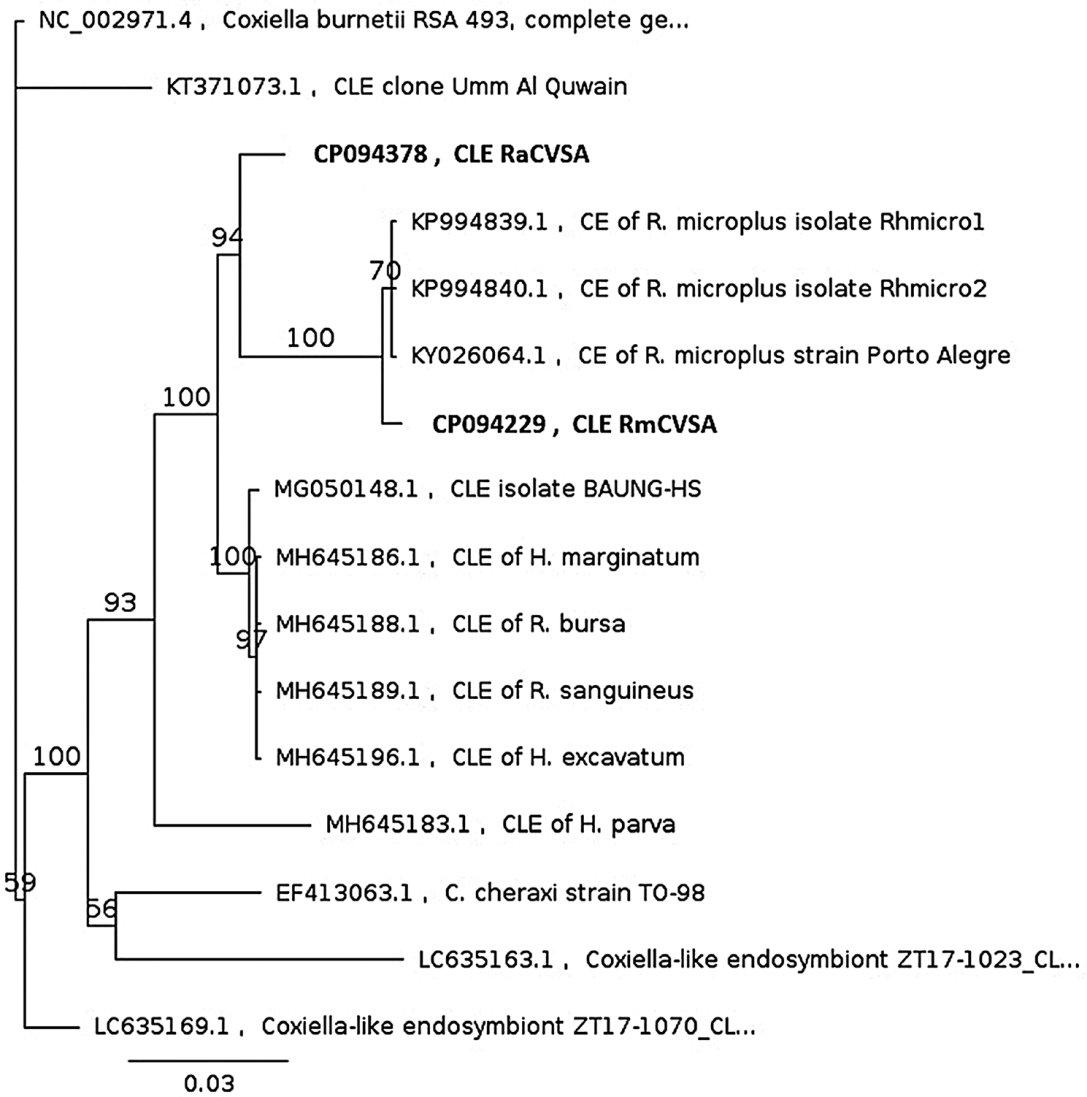
Bayesian inference phylogenetic analysis of 16 S rDNA sequences obtained from RmCVSA_CLE and Ra_CVSA_CLE (bolded) when compared to CLE sequences available from GenBank. *C. burnetii* served as the outgroup

The contigs that were generated from the genome assemblies that mapped to the sequences in the bacterial 16 S and *Coxiella* spp.-generated databases were removed from the tick genome assemblies. These contigs, targeting the 16 S region primarily, would remove reads assembled with an 16 S target region, resulting in non-16 S-realted bacterial target contigs obtained through horizontally gene transfer, to remain part of the tick genome assemblies. All FASTQ base called reads were subsequently mapped against the *Coxiella* spp. database, and mapped reads were used in a Flye assembly to generate a single circular bacterial contig from each dataset. The RmCVSA_CLE and RaCVSA_CLE genome assemblies achieved a coverage of 1,522X and 106X, respectively. Molecular phylogenetic analysis of the 16 S rDNA sequences indicated that RmCVSA_CLE clustered with three other *R. microplus*-derived CLEs to form the Rm_CLE clade and that RaCVSA_CLE shared a common ancestor with the Rm_CLE clade (Fig. 8).

**Fig. 9:**
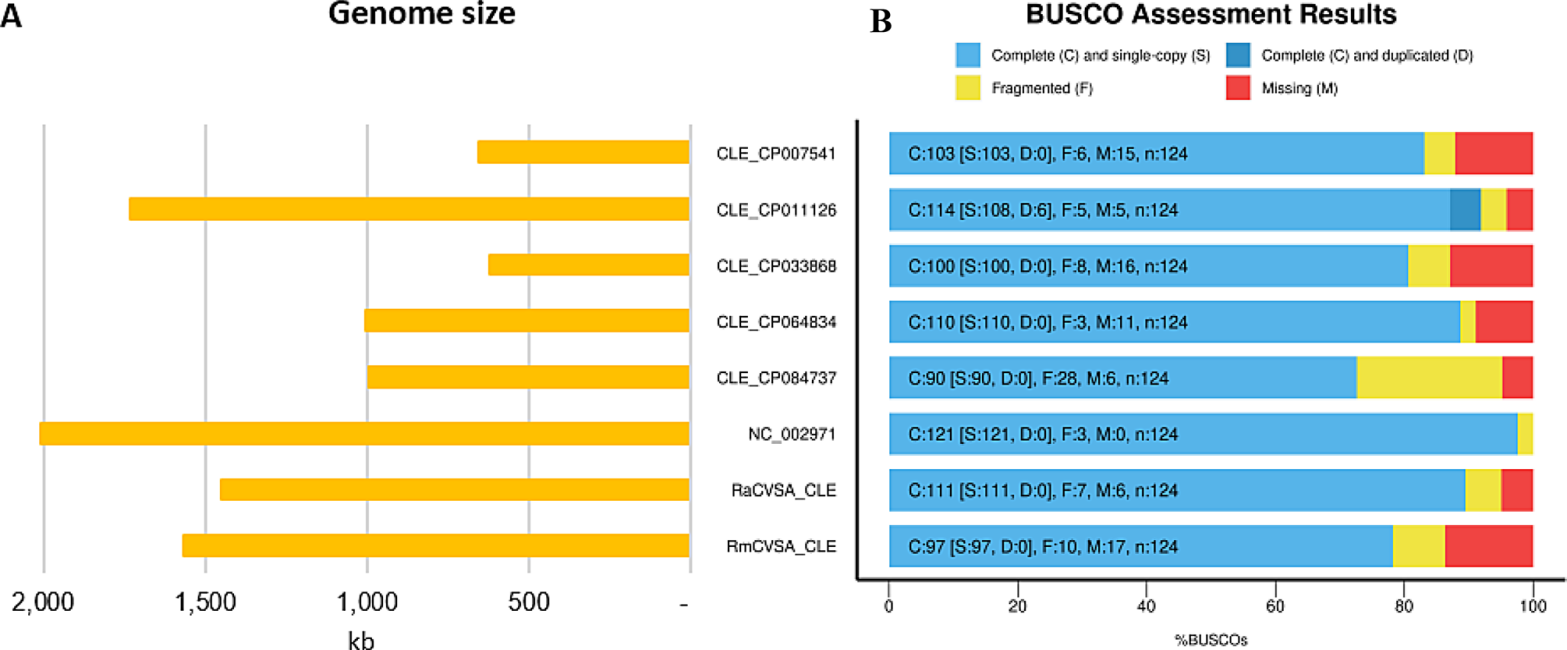
Genome size comparison (**A**) and BUSCO assessment (**B**) of *C. burnetii* (NC_002971) and other CLEs from ticks

The polished RmCVSA_CLE and RaCVSA_CLE single contig genome assemblies were compared to other CLE genomes and subjected to BUSCO analysis to assess the genome assembly and compare the BUSCO scores with other CLEs as well as the free-living *C. burnetii* genome (Fig. 9).

BUSCO results indicated that CLE genomes (CLE_CP007541 and CLE_CP033868) represented only 31% of the *C. burnetii* genome; however, they contained 80% of the complete targets in the BUSCO bacterial database (Fig. 9). RmCVSA_CLE and RaCVSA_CLE exhibited genome reductions of 22% and 28%, respectively, when compared to *C. burnetii*. BUSCO analysis revealed that even though RmCVSA_CLE had a larger assembled genome than RaCVSA_CLE, more BUSCO targets were missing in the genome with 10X more sequence coverage (17 BUSCOs missing in RmCVSA_CLE compared to 6 missing in RaCVSA_CLE). This was also the case after genome annotation using the RAST server (Table 7), followed by comparative analysis using the curated SEED database.

**Table 7:** RAST generated data for *C. burnetii* CbuG Q212, RmCVSA_CLE and RaCVSA_CLE

	C. burnetii	RmCVSA_CLE	RaCVSA_CLE
Size	2,008,870	1,566,152	1,450,318
GC Content %	43	31	38
Number of Subsystems	298	152	187

**Fig. 10:**
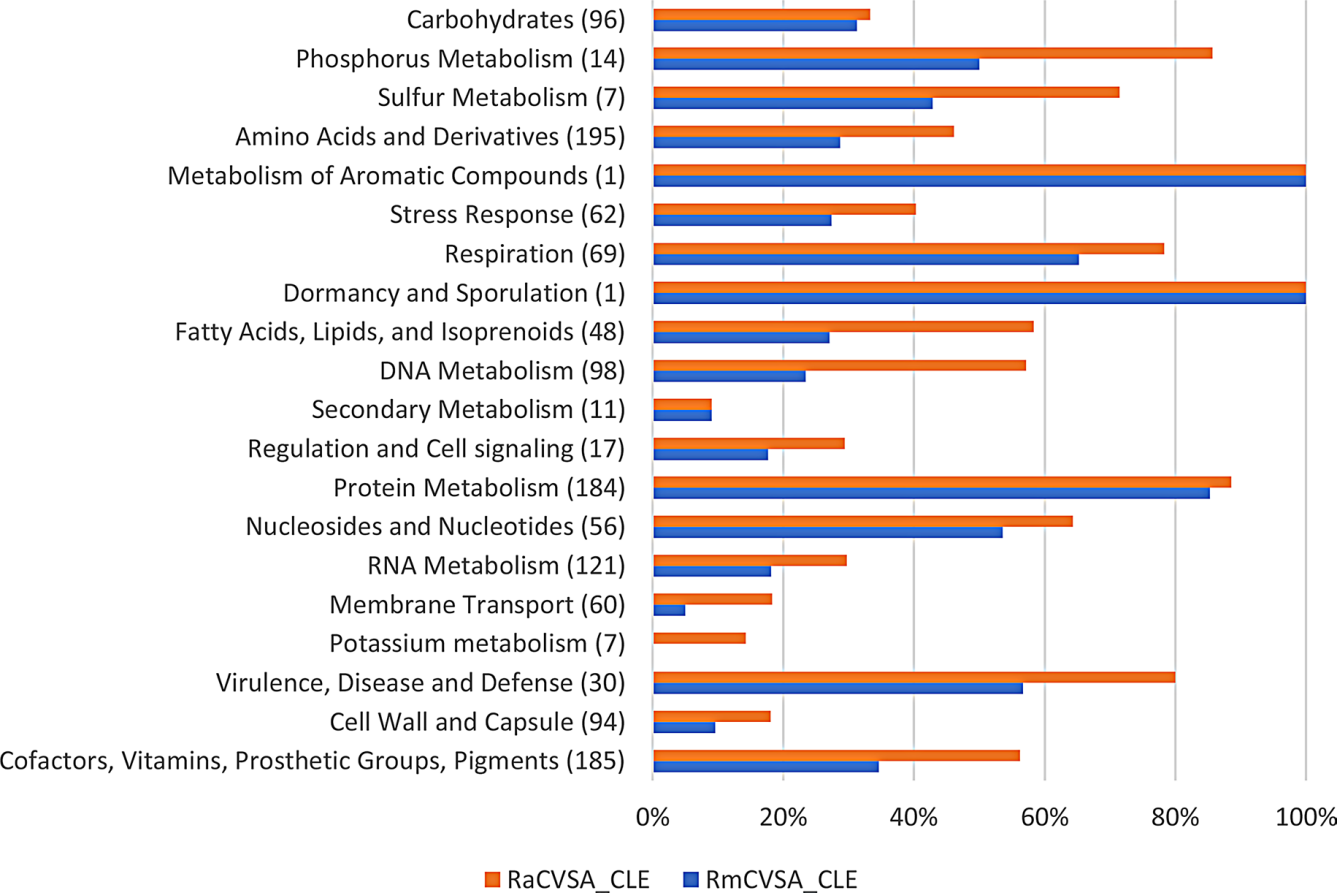
RAST annotation and SEED-based comparative analysis of RmCVSA_CLE and RaCVSA_CLE genomes relative to the *C. burnetii* genome. The number of subsystems present in the *C. burnetii* genome is indicated in brackets following the subsystem category

Comparative analysis between *C. burnetii*, RmCVSA_CLE and RaCVSA_CLE using SEED data revealed that the CLE genomes contained < 63% of the subsystems assigned to *C. burnetii*. Expression of the RmCVSA_CLE and RaCVSA_CLE subsystems as a percentage of the assigned *C. burnetii* subsystems provides insights into the effect of the observed genome reductions on the impact of biological functionality (Fig. 10).

Functional biotin synthesis subsystems (part of the Cofactors, Vitamins, Prosthetic Groups, Pigments group in Fig. 10) were identified and assigned to both RmCVSA_CLE and RaCVSA_CLE, and these subsystems fit within the critical role that the tick CLE plays as a nutritional endosymbiont, providing supplementary B vitamins needed for survival [21].

## Discussion

In this study, we aimed to address the challenges associated with assembling large and complex genomes, which are often excessively costly due to factors such as sequence data generation, computational resources, sample preparation, and data storage. The primary aim was to demonstrate the feasibility of generating assemblies for two cattle tick species, *R. microplus* and *R. appendiculatus*, using a cost-effective approach focused on Oxford Nanopore sequencing technology.

During the study, we employed cost-effective methods using readily available laboratory reagents, consumables, and equipment for tick DNA isolation. The isolated DNA exhibited HMW and yielded sufficient quantities for all necessary sequencing, all derived from a single isolation procedure starting with 1.25 g of fertilized tick eggs. We utilized agarose gel electrophoresis for DNA quality assessment using a commercially available high-range DNA ladder as a reference standard. Although it is not an optimal method for assessing DNA with sizes exceeding 50 kbp, it is one of the most basic techniques used in any standard molecular biology laboratory and requires no additional upfront investment in expensive specialized equipment. To prepare the libraries, the isolated DNA was subjected to a low-cost, low-technology shearing approach using a 29G needle and a 1 ml disposable pipette tip to generate starting material for library construction. Sequencing of the libraries resulted in long, lower yielding Nanopore sequence reads for the libraries prepared from the P1000 sheared DNA, while the shorter, high yielding sequence reads were obtained from the 29G sheared DNA libraries. Occasional rapid pore blockage was observed for the libraries during sequencing, especially those derived from the P1000 sheared tick DNA. However, DNase I treatment of the flow cell and reloading resulted in a > 4-fold increase in yield from a single flow cell. Using this optimized approach, we successfully generated sufficient sequence data of different lengths, using a total of 10 flow cells for the final *de novo* assembly of each tick genome. The current availability of the PromethION 2 Solo starter pack will increase cost efficiency and throughput, whereas the availability of the PromethION option in 2021/2022 would be more than two orders of magnitude more capital intensive when compared to the MinION option.

Base calling of Nanopore fast5 files generated by the MinION device can be performed on a local computer using CPU processing; however, this can be prohibitively slow (especially when using the more accurate base calling algorithms) and requires large numbers of cores. This obstacle can be overcome with the use of GPUs, which significantly increases base calling speed but requires specialized hardware and software. Cloud services, such as AWS, offer GPU instances; however, fast5 files are large, ranging up to 100 GB in size per flow cell. Uploading such large datasets to the cloud can be time consuming, especially with limited bandwidth, and the large storage space needed can become expensive. Utilizing a local computer with a dedicated GPU to convert the fast5 files to FASTQ files resulted in an approximate 8-fold reduction in file size for uploading to AWS for *de novo* genome assembly. The utilization of a local machine also facilitated the evaluation of different base calling models (HAC and SUP) as well as uploading and analysis of the base called data.

The *de novo* genome assembly of large (> 2 Gbp) and complex (long repeat rich) genomes requires access to high-performance computing (HPC) due to the extensive data analysis and processing needed, especially when no reference genome is available as a scaffold. As new entrants to genomics, we lacked access to any local HPC resources and had to make use of alternative resources. Amazon Web Services was selected as a cloud-based alternative since the large datasets generated for the study could be stored and processed at a reasonable cost, without making any capital investment, and it provides a pay-as-you-use option. Notably, AWS offers spot instances, which come at discounted hourly rates, drastically reducing the overall cloud computing costs (up to 90%), albeit there is a chance for the instance to be interrupted when these discounted resources are needed by “on-demand” instances. In this study, we exclusively used AWS spot instances to achieve cost-effective cloud computing.

The Shasta and Flye assembly software tools [24, 25], running on AWS spot instances, were evaluated based on the ability to perform *de novo* genome assembly using nanopore-generated DNA sequence data from *R. microplus*-derived DNA libraries. A well-known error commonly found in Nanopore data is homopolymer errors, specifically deletions within these regions [29]. The Shasta assembler is specifically optimized to mitigate these errors by compressing homopolymers during the data storing phase and shows significantly faster processing speeds compared to most long-read assemblers. Shasta reduces the overall cost of *de novo* assembly of large complex genomes with a > 270-fold reduction in assembly cost compared to Canu [25]. Since Shasta uses an in-memory computing-driven algorithm, it requires a large amount of RAM, typically between 1 and 2 TB, to assemble a human genome, depending on the coverage [25, 30]. The Shasta requirements for the *de novo* assembly of human genomes were used as a guide to select the parameters for AWS-based *de novo* assembly of the Nanopore-generated tick genome sequences. Flye was shown to generate more accurate and contiguous assemblies of genomes with repetitive regions compared to other tools, such as Canu and MaSuRCA [24]. Additionally, the runtime of Flye was notably faster than that of the aforementioned tools, and the polishing step included in the algorithm has the potential to reduce the error rates of nanopore reads [24]. Assembly statistics and BUSCO analysis of the assembled genomes indicated that the Shasta assemblies were superior to the Flye assemblies (Supplementary Table 1; Fig. 2) but were 30 times faster in generating the initial assembly (Table 3). The low computing cost associated with our current assembly workflow (USD < 150 per final assembly) allowed us to perform a thorough comparison between the HAC and SUP base called data and evaluate the different assemblies using BUSCO scores as a measure of completeness. *Rhipicephalus microplus* and *R. appendiculatus* genome assemblies based on the HAC base called data proved to be more complete based on BUSCO scores compared to the SUP base called data (Fig. 2). Comparative analysis of the three *R. microplus* nuclear genomes based on assembly statistics (Table 5) and BUSCO analysis (Fig. 3) showed that our current workflow generated a genome assembly comparable to the current published assemblies, providing access to inter- and intraspecies repeat content present in the tick genomes (Fig. 7).

Jia et al. (2020) published data on the genome sequence of *R. microplus* as part of six tick species genomes that were sequenced and assembled. Their current reference *R. microplus* assembly (ASM1333972v1) was based on the PacBio reads using four *de novo* assemblers, and the best assembly was chosen based on optimal continuity and completeness. Thereafter, Illumina reads were used for polishing and error correction. Finally, Hi-C reads were used for scaffolding. The publication lacked information on computational and sequencing costs, making any direct comparisons impossible. Guerrero et al. (2021) recently published an *R. microplus* (ASM1343599v1) assembly, with the initial idea of using a combination of Illumina and PacBio reads, where the Illumina reads would be used to correct the long PacBio reads. They, however, stated that the computational resources needed for this approach could not be accessed, resulting in an assembly that only used the PacBio reads in the Canu pipeline. This assembly ran for approximately 25 consecutive days on a reserved node with 352 cores and 12 TB of RAM. The results from this study demonstrate that exclusively relying on Oxford Nanopore sequencing, coupled with optimized bioinformatics workflows and cost-effective cloud computing resources such as AWS spot instances, can yield high-quality genome assemblies. These genomes, when compared to previously published tick genomes that used multiple sequencing platforms and resource-intensive bioinformatics workflows, show comparable BUSCO scores. This highlights the potential for low-resource environments to engage in genomics research without the need for expensive capital investments.

Regarding the annotation of RaCVSA and RmCVSA, having a reference available for annotation proves advantageous in terms of computational cost and time. Utilizing a reference drastically reduces the time required to annotate a genome compared to running BRAKER3, although resulting in a less complete annotation. If a reference is unavailable, as in the case of *R. appendiculatus*, BRAKER3 was used. A major advantage of BRAKER3 is its automated pipeline, which provides an efficient solution for genome annotation in the absence of a reference. However, BRAKER3 requires transcriptomic data to run and entails setting up several dependencies through local installation, which may pose a challenge for less experienced users [1, 31]. Despite this, BRAKER3 produces more complete annotations and encounters fewer formatting issues with the General Feature Format (GFF) file when submitting to GenBank compared to the Liftoff reference-based annotation, which we ultimately excluded from further analyses due to these formatting challenges.

The final annotated RmCVSA assembly (RmCVSA SR) consisted of 60,935 genes and 32,263 functional annotations, with 30,501 of the predicted genes being supported by both the transcriptomic and ab initio approaches. Other published *R. microplus* genomes, such as RmGuerrero, has not been annotated, whereas the GenBank annotation for RmJia includes 29,870 genes, of which 29,866 are protein-coding [10]. For comparison, the *I. scapularis* reference genome comprises 38,656 genes, of which 26,659 are protein-coding [32]. Notably, there is currently no annotated assembly available for *R. appendiculatus*, highlighting the significance of our findings in expanding the genomic resources for this species.

The high number of predicted genes observed in our annotations may have been influenced by the use of different gene prediction methods compared to other studies, since bioinformatic approaches evolve rapidly to include and predict additional genes solely by the ab initio approach, which may lack corresponding RNA-based evidence for support. Retaining these genes in genome annotations is important since they may be validated by future experimental data as new transcriptomic data become available, ensuring a comprehensive and inclusive annotation. Current available transcriptome and proteomic datasets might not be comprehensive enough to detect all expressed genes, especially those with low expression levels. Including unsupported genes allows for the possibility that they are present but undetected due to technological limitations [33]. Additionally, some genes might be functionally redundant and therefore not expressed under normal conditions but could be expressed under specific environmental conditions [33]. Furthermore, some unsupported predictions might represent pseudogenes, regulatory elements, or other genomic features that, while not coding for proteins, are still important for genome function and architecture [34].

Both the RaCVSA and RmCVSA annotations showed a high level of completeness based on the gene predictions which was evaluated by BUSCO in protein mode.

An additional aim of this study was to address the challenge of sequencing and assembling highly AT-rich mitochondrial DNA, given the error rate associated with nanopore sequencing of homopolymer regions. While the sequencing and assembly produced accurate mitochondrial genomes, we encountered limitations associated with imperfect base calling in homopolymer regions exceeding 10 bases, which was slightly improved with Q20 + sequencing. The RmCVSA mtDNA genome (based on the genome generated from the genomic sequencing as well as the separate mtDNA genome generated from the overlapping long PCR amplicons) had five tandem repeat DNA sequences present, containing the 3’ end of NAD1 and Glu-tRNA. This exact repeat structure was also present in an *R. microplus* isolate from Australia, but unfortunately, no complete mtDNA genome was sequenced for a complete comparison [35]. All five of these tandem repeat sequences could not be detected in any of the complete mtDNA genomes in GenBank, where at most four repeat sequences could be detected. The mtDNA sequence exhibiting the highest nucleotide identity toward RmCVSA was KY457541, which also originated from the same *R. microplus* tick colony in Clinvet, South Africa. It was sequenced using Illumina short read technology, most likely resulting in bioinformatic truncation of the repeat unit, since the fifth repeat yields a 649 bp sequence that exceeds the paired read length overlap needed for short-read sequencing. The RmCVSA mtDNA genome was 99.9% identical to *R. appendiculatus* mtDNA genomes originating from South Africa (KY457535 and KY457536) and 97.4% identical to the mtDNA genome of an *R. appendiculatus* isolate originating from Kenya (NC_052829), with the lower identity attributed to the presence of a repeat region within control region 2.

This study also successfully employed the same sequencing data to assemble and annotate genetically distinct CLE genomes for each tick species. Gene loss is a common phenomenon observed in symbiotic bacteria, as host tissues offer a relatively stable environment where nonessential genes are no longer under selective pressure, leading to genome reduction [36]. Phylogenetic 16 S rRNA analysis indicated that RmCVSA_CLE belonged to the *R. microplus* CLE clade, where RaCVSA_CLE shared an ancestor with the *R. microplus* CLE isolates but was genetically distinct (Fig. 8). This observation is interesting since both isolates are artificially reared in the same laboratory using different host species. The > 37% reduction in detectable RmCVSA_CLE and RaCVSA_CLE subsystems indicates the impact of the symbiotic relationship when compared to a free-living *C. burnetii* genome, taking into consideration that both endosymbionts contained > 80% of the single copy bacterial ortholog targets evaluated during the BUSCO analysis. The absence of > 80% in cell wall and membrane transport subsystems suggests evolutionary adaptation to a more protective endosymbiont environment, while the presence of an intact biotin synthesis pathway in both isolates supports the nutritional support for the host in this endosymbiotic relationship.

## Conclusion

Access to reliable genome data accelerates progress in the field of genomics, including biomining, genetic engineering and genotype–phenotype association studies. The initial draft genome for *R. microplus* [11] yielded limited success in the identification of acaricide resistance target genes, and the genes that could be identified resulted in amplification failure of DNA from a South African *R. microplus* isolate using primers designed on the draft genome sequence data (based on our unpublished observations). It also revealed the presence of complex repeat regions, requiring long-read sequence technology to allow direct sequencing of the *R. microplus* nuclear genome without relying on repeat depletion strategies and allowing for spanning of the repeat regions, resulting in a more accurate assembly. Nanopore sequence technology using the MinION device with a simple workflow was the only long-read technology that we could acquire within our limited budget when the project started in the last quarter of 2021. The relatively low capital investment of USD 3,250 facilitated full-time access to a local device and allowed base calling optimization and rebase calling using updated base calling algorithms on the local computer, while reducing the file size allowed more efficient uploading. This, in turn, afforded the flexibility to optimize the library preparation and sequencing strategy in real time. For less than USD 10,000, utilizing nanopore sequencing technology, we were able to generate sufficient genome coverage and read N50 values, resulting in the first African derived *R. microplus* annotated genome that was on par with the recently published *R. microplus* genomes from Asian and American origin. Initial planning for using Illumina short read sequencing by a South African service provider would have resulted in a sequencing cost of approximately USD 44 300 per genome (based on the 2021 quote and an exchange rate of ZAR 16.47 to the dollar), which would have been cost prohibitive for the study completion. The assembly of large and complex genomes usually requires substantial computing resources, which are not always available or feasible in smaller laboratory setups. The relatively large datasets that were generated could easily be stored and processed at a reasonable cost with AWS, which served as our sole bioinformatic computing resource. Our final assembled genomes exhibited statistical and bioinformatic data quality comparable to newly generated genomes that employed multiple sequencing technologies and computing resources, typically accessible only to multidisciplinary teams involved in large-scale genomic research projects [10, 12]. The bioinformatics workflow employed to generate the *R. microplus* draft genome was used to generate the first publicly available annotated *R. appendiculatus* nuclear genome that exhibited the same characteristics in terms of assembly statistics and genome completion assessments.

The generated sequence data facilitated the assembly of complete *Coxiella*-like endosymbiont genomes for both *R. microplus* and *R. appendiculatus*. These divergent CLE genomes exhibited differential genome reduction characteristics and properties, despite both isolates being reared and maintained in the same laboratory environment, indicating that the CLEs are species specific.

The > 80% AT-rich mitochondrial genomes could also be generated using a long PCR amplicon sequencing strategy in combination with Q20 + nanopore sequence technology.

This study provides evidence of a low-cost alternative approach to independently sequence and accurately assemble relatively large, highly repetitive, and complex genomes, without the capital-intensive investment usually needed for genome sequencing. The *R. microplus* data set generated in this study served as reference for accurate identification, amplification and sequence analysis if historical targets implicated in acaricide resistance that would not be possible based on data available in 2021 [37].

## Methods

### Genomic DNA isolation and library preparation

*Rhipicephalus microplus* larvae (RmCVSA, originally isolated from the Eastern Cape, South Africa in 2018) were placed on cattle, and approximately 21 days later, detached engorged females were collected. Ten engorged females were placed in petri dishes in a controlled environment (~ 26 °C, 75% relative humidity) to allow oviposition. After approximately 10 days, all females completed oviposition, and eggs were left to develop for approximately 30 days and frozen at -80 °C until use.

Unfed *R. appendiculatus* adults (RaCVSA, originally isolated from Mpumalanga, South Africa in 2016) were placed on a rabbit to feed, and approximately 10 days after infestation, detached engorged females were collected. Engorged females were placed in petri dishes in a controlled environment (~ 26 °C, 75% relative humidity) to allow oviposition. After approximately 12 days, all females completed oviposition, and eggs were left to develop for approximately 28 days and frozen at -80 °C until use.

Eggs (1.25 g) from approximately 20 females were ground to a fine powder under liquid nitrogen and transferred to a 50 ml centrifuge tube containing 30 ml lysis buffer (10 mM Tris-HCl pH 8, 400 mM NaCl, 2 mM EDTA pH 8). Proteinase K solution [5 ml containing 1% (v/v) SDS, 5 mg/ml proteinase K] was added to the 50 ml centrifuge tube followed by rotation at 10 revolutions per minute (rpm) while incubated at 56 °C for 16 h. Ten milliliters of Tris-HCl-equilibrated phenol (pH 8.0) was added to the lysate and rotated for 10 min at 10 rpm at room temperature. The mixture was centrifuged at 3000 x *g* for 30 min at room temperature, and the aqueous phase was extracted for a second round with 5 ml phenol as described above. The resulting aqueous phase was subsequently extracted twice with 10 ml of chloroform with centrifugation at 3000 x *g* for 30 min in between. The chloroform-extracted aqueous phase was supplemented with 0.4 volumes of 5 M NaCl and inverted 10 times end-over-end, followed by the addition of two volumes of absolute ethanol. The tube was inverted 10 times end-over-end and incubated at 20 °C for 5 min. DNA was spooled out using a 1 ml pipette tip, followed by washing with 40 ml 70% (v/v) ethanol and inverting the tube 10 times end-over-end. The DNA was collected by spooling using a 1 ml pipette tip and allowed to drip dry. The DNA was placed into a new 50 ml tube, and visible traces of liquid were removed by pipetting. The uncapped 50 ml tube was placed in an incubator at 37 °C for 10 min, followed by the addition of 5 ml TE buffer (pH 8) containing 20 µg/ml RNase A and incubation at 20 °C for 3 h on a rotator at 10 rpm. Agarose gel electrophoresis [0.5% (m/v) agarose in 1 x TAE containing 1 x SYBR Safe stain electrophoresed at 3.5 V/cm for 2 h] was used to evaluate the size and integrity of the isolated DNA, and spectrophotometric analysis was used to quantify the isolated DNA. The isolated DNA was stored at 4–8 °C until use.

The ONT SQK-LSK109 library prep kit was used to prepare long-read libraries. The Long-read club Mountain protocol (https://www.longreadclub.org/mountain-protocol/) was used for library preparation, making use of a bead clean-up approach. A total of 10 µg HMW DNA was used as input material. The DNA was subjected to two different shearing strategies. Strategy 1 entailed shearing the DNA 15 times through a 29G needle to generate high yield sequencing libraries, and in strategy 2, the DNA was subjected 15 times passing through a 1000 µl Nest disposable pipette tip to generate lower yield longer read libraries. The sheared DNA was purified using AMPure XP beads as per the Mountain protocol, and 400 ng of the final library was loaded onto each of the MinION flow cells (R9.4) and allowed to sequence for 96 h. Strategy 2 resulted in significant pore blockage and was subjected to a DNase I wash for 30 min every 12 h, followed by priming and reloading of the flow cell.

The DNA from *R. appendiculatus* was also subjected to fragmentation and library preparation as described above. A single 29G sheared library was also prepared using the SQK-LSK112 library prep kit and sequenced in a single run on the R10.4 flow cell (Q20+) to generate higher accuracy data for mitochondrial DNA assembly assessment.

### Mitochondrial DNA amplification and sequencing

Primers based on the *R. appendiculatus* mtDNA (KY457536) and the *R. microplus* mtDNA (KP143546) genomes were designed to amplify the complete mtDNA genomes in 2 long overlapping sections (Table 8). Target amplification was performed using LongAmp^®^*Taq* DNA mastermix (NEB) in a 25 µl final reaction volume containing 0.4 µM of each primer pair and 50 ng HMW genomic DNA. Thermal cycling was performed using an initial denaturation of 30 s at 94 °C followed by 30 cycles of 94 °C for 15 s, 52 °C for 1 min, 65 °C for 11 min and a final elongation of 65 °C for 10 min. PCR products were analyzed using agarose gel electrophoresis.

**Table 8:** Oligonucleotide primers used to generate long amplicon overlap PCR products for MtDNA sequencing

Forward primer (sequence in 5’-3’ orientation)	Reverse primer (sequence in 5’-3’ orientation)	Expected size (annotation)
Ra_mtDNA_ctrl-2 F (GCCTATGCTATTCTTCGATCAATTCC)	Ra_mtDNA-2R (TATAAATTAAGGACAAGAAGACCCTATG)	8 001 bp (Ra_L1)
Ra_mtDNA-2 F (CTGTACGACTAACTGCTAATATAATTAG)	Ra_mtDNA_ctrl-1R (GTATGAACCCAATAGCTTAGAAATTAG)	10 651 bp (Ra_L2)
Rm mtDNA-1F_1 (CCTATTAACAATTCCTTTAATAATAACTC)	Rm mtDNA-4R_1 (AGCTTGAACTACAAAATATGTAATTATAGC)	11 346 bp (Rm_L1)
Rm mtDNA-3 F (GTGATAACACTCAAAGTTATTTGCAC)	Rm mtDNA-1R (GTACTTACGTGCGAATACGATATG)	12 042 bp (Rm_L2)

PCR products were pooled, purified, and subjected to library preparation using the SQK-LSK112 library prep kit and sequenced in a single run on the R10.4 flow cell to generate higher accuracy data for mitochondrial DNA assembly assessment. Ambiguities between the reference and assembled mtDNA sequences were amplified and sequenced on both strands using Sanger sequencing to confirm sequence accuracy.

### Base calling of sequence data

Fast5 files generated by the MinION device using the R9.4 flow cells were basecalled on a local computer with an NVIDIA GeForce RTX 2080 Ti graphics card. Basecalling of DNA sequences was performed with Guppy GPU base calling. Initially, Guppy version 4.0.14 (Supplementary Table 3) was used with the HAC model and the dna_r9.4.1_450bps_hac.cfg config file, and filtering parameters were set to “--min_qscore 10” and “--qscore_filtering”. Base calling was later redone with a newer version of Guppy, version 5.0.11 and the SUP model with the dna_r9.4.1_450bps_sup.cfg config file and filtering parameter “--min_qscore 10”. Fast5 files generated from the R10.4 flow cells and the SQK-LSK112 library kit were base called using Guppy, version 5.0.11 and the SUP model with the dna_r10.4_e8.1_sup.cfg config file and filtering parameter “--min_qscore 10”.

After basecalling, NanoStat was used to calculate a summary of various statistics of the reads. Thereafter, NanoFilt was used to filter out any reads with a score lower than Q10.

### Genome assembly

The Shasta genome assembler was used to perform the *de novo* assemblies of the tick genomes (Supplementary Tables 1 and 3). For each tick species, two *de novo* assemblies were performed. The first assembly was based on a minimum read length of 1,000, whereas the second assembly had a minimum read length of 10,000. The Q10-filtered FASTQ reads were converted to FASTA format using the “seq” command from Seqtk software. Thereafter, the Q10 reads were *de novo* assembled using the Shasta assembler. The “Nanopore-Sep2020.conf” config file was used with default parameters, except for the “minimum read length” parameter, which was set accordingly. For comparison, the Flye assembler [24] was used to generate a *de novo* assembly of *R. microplus* using default parameters. The assemblies ran on Amazon Web Services (AWS) using the *x1.32xlarge* instance, which consists of 64 cores (128 vCPUs) and 1,952 GB RAM on a Linux machine using spot instances.

### MarginPolish

Raw reads were mapped back to the Shasta and Flye assemblies using Minimap2 (Supplementary Table 3) with the parameter “–ax map-ont”. The generated SAM files were then converted to BAM format, sorted, and then indexed using SAMtools. The resulting BAM files were used as input to polish the assemblies. For each assembly, the resulting BAM file was used as input for the graph-based assembly polisher MarginPolish. MarginPolish was performed using AWS instance *r4.8xlarge*, which has 16 cores (32 vCPU) and 244 GB RAM on a Linux machine using a spot instance. Default parameters were used for polishing.

### Genomic contig filtering

BLAST databases, based on available bacterial mitochondrial sequence and bacterial 16 S sequences were created using NCBI’s command-line tool, “makeblastdb” to identify and remove all known mitochondrial and bacterial contigs generated during the assembly, respectively. Polished contigs from all tick assemblies were aligned to these databases using “blastn” with default parameters to identify and filter out any known non-tick-related contigs.

### Bacterial genome assembly

The tick assemblies were first aligned to the NCBI 16 S database using *BLASTN* to identify bacterial contamination present in the assemblies for each tick species.

The bacterial database used for *R. microplus* and *R. appendiculatus* was built using complete bacterial genomes available on the NCBI for *Coxiella burnetii* and other *Coxiella*-like endosymbionts. For each assembly, all contigs were aligned to the bacterial database using *BLASTN.* The identified bacterial contigs were discarded, as Shasta could not fully assemble the contaminating bacterial genomes. These datasets were then *de novo* assembled using the Flye assembly program with the “--nanoraw” and “--meta” parameters and “--genome-size” set to 2 m. To obtain bacterial genome coverages, raw reads from each tick species were mapped to the relevant obtained bacterial genomes using Minimap2 with the parameter “–ax map-ont”. Thereafter, BEDTools “genomecov” was used with default parameters to calculate coverage across the genomes.

The assembled bacterial contigs were polished using two rounds of Medaka polishing with default parameters. The polished contigs were then annotated using the RAST annotation pipeline [38] and comparative analysis was performed using SEED [39].

For taxonomic classification of the assembled bacterial contigs, a phylogenetic tree was constructed based on 16 S rDNA sequences using “MRBAYES” (HKY85 substitution model; 1,000,000 chain length; 5 heated chains; 1,000 subsampling frequency; 100,000 burn in) with *Coxiella burnetii* NC_002791.4 16 S used as the outgroup.

### Mitochondrial genome assembly

A mitochondrial database was built for each tick spp. using relevant available *Rhipicephalus* mitochondrial genomes available on the NCBI. After bacterial extraction, the remaining genomic DNA contigs from the polished assemblies were aligned to the relevant mitochondrial databases for each spp.

Reads obtained from RaCVSA and RmCVSA mtDNA generated using the Q20 + kit were mapped to the known *R. appendiculatus* and *R. microplus* mitochondrial DNA reference genomes, and all mapped reads were extracted and *de novo* assembled using the Flye assembler with the “-- nanoraw” and “--genome-size” set to 15k. The contig generated was compared to the reference mtDNA genomes using multiple sequence alignment (MAFFT) analysis.

For RaCVSA, identified mitochondrial contigs were extracted and assembled into a single contig using the Flye assembler with the “--nanoraw” and “--meta” parameters and “--genome-size” set to 15k. All mitochondrial genomes were then polished with three rounds of Medaka polishing and annotated in Geneious Prime based on sequence homology using a database of related fully annotated mitochondrial genomes as references.

Available complete *Rhipicephalus* spp. mitochondrial genomes were downloaded from GenBank and used for phylogenetic analysis. Protein-coding genes were extracted from each assembly, concatenated, and aligned using MAFFT followed by phylogenetic tree construction using “MRBAYES” with the HKY85 substitution model.

The RmCVSA and RaCVSA mtDNA genomes were annotated using the MITOS web server and submitted to GenBank.

### PurgeHaplotigs

Haplotigs were removed using the Purge Haplotigs pipeline (Supplementary Table 3). All raw reads were mapped to the cleaned assemblies to generate BAM files using Minimap2. The BAM files were then sorted and indexed using SAMtools. The Purge Haplotigs pipeline was executed on a Linux machine on AWS using the *r5a.4xlarge* instance with 16 vCPUs and 128 GB RAM.

### QuickMerge

For each tick species, the two-phased assembled genomes were “merged” to generate a more contiguous assembly using *quickmerge* (Supplementary Table 3). The following steps were run on a Linux machine on AWS using the *r5a.4xlarge* instance with 16 vCPUs and 128 GB RAM and cost USD 0.2765 per hour. First, the “nucmer” command from MUMmer was used with parameter “-l 100” to align the two assemblies, followed by the “delta-filter” command to identify the best alignments. For RmCVSA, delta-filter parameters were set to “-r -q -l 100000”, whereas for RaCVSA, they were set to “-r -q -l 10000”. MUMmer version 4 was used since it allows multithreading, which decreases the run time.

Quickmerge requires a query and a reference assembly. For RmCVSA, the 10,000 assembly was set as a reference and 1,000 as a query, and vice versa for RaCVSA.

**Fig. 11:**
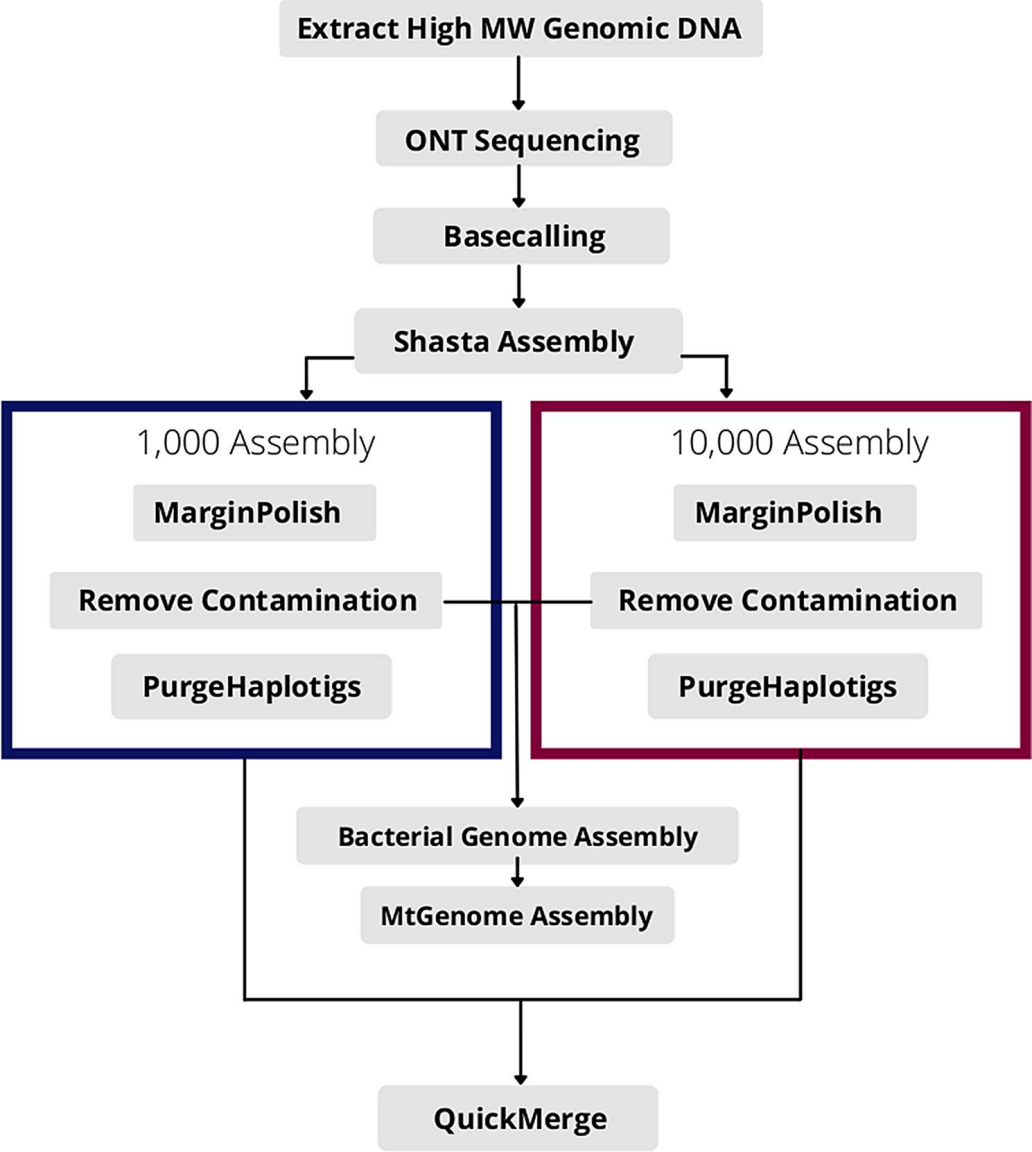
Genome sequence generation and assembly workflow used during the study to convert tick DNA-derived sequences into high-quality assemblies

A summary of the genome sequence generation and assembly workflow used to generate high-quality genomes from isolated DNA is presented in Fig. 11.

### Genome coverage

To calculate the genome coverage of the RmCVSA and RaCVSA assemblies, raw reads were mapped to the final assemblies using Minimap2 with the parameter “–ax map-ont”. Thereafter, BEDTools “genomecov” was used with default parameters [40].

### Assembly statistics

The software program *assembly stats* was used to summarize and visualize the assembly statistics of the genome assemblies. Plots were generated using https://github.com/rjchallis/assembly stats.

### BUSCO

The completeness of the final assemblies was assessed using Benchmarking Universal Single-Copy Orthologs (BUSCO) in genome mode [41]. All available GenBank assemblies for *R. microplus* (Table 5) were evaluated using BUSCO version 5.6.1 with the arthropoda_odb10 BUSCO lineage and MetaEuk gene predictor. The current Arthropoda database (arthropoda_odb10.2024-01-08) contains 1013 highly conserved genes. Another software tool, compleasm, was used to assess the assembly completeness of the *R. microplus* genomes. In contrast to BUSCO, compleasm utilizes Miniprot for predicting protein-coding genes in eukaryotic organisms. This gene predictor offers a shorter runtime and enhanced accuracy in detecting splice junctions and frameshifts [42].

The bacterial endosymbiont BUSCO analysis utilized the bacteria_odb10 database, encompassing 124 conserved genes, and the prodigal gene predictor.

### Repeats

Repeat families were identified in each assembly using *RepeatModeler* (Supplementary Table 3). This was run on the Genome Sequence Annotation Server (GenSAS), a web-based platform that provides a pipeline for whole genome structural and functional annotation for eukaryotes and prokaryotes. Repeat families for *R. microplus* GenBank assemblies (RmJia and RmGuerrero; Table 5) were also re-evaluated on GenSAS using *RepeatModeler* to allow a direct comparison.

### Annotation

We employed RepeatModeler2 with default parameters [43] to perform *de novo* identification and compilation of sequence models representing all unique transposable element (TE) families spread across the RmCVSA and RaCVSA genomes. The generated *de novo* repeat libraries were used as repeat databases to mask the genomes using RepeatMasker [44].

For RaCVSA, SR sialo transcriptomic data retrieved from GenBank (SRR2568016-19) [45] were used along with the Arthropoda protein database sourced from OrthoDB v11 [46] in the BRAKER3 pipeline [47–51]. Within BRAKER3, the GeneMark-ETP pipeline was utilized to assemble the transcriptomic sequences to the genome using StingTie2 [52]. Subsequently, the assembled transcripts underwent analysis by GeneMark-ST for the prediction of protein-coding genes, which were then matched against the provided protein database. Based on the outcomes from GeneMark-ETP, AUGUSTUS was employed to further predict genes, while TSEBRA integrated the predictions generated by both GeneMark-ETP and AUGUSTUS to generate the final annotation [53–57]. TSEBRA removes redundant predictions, filters out predictions that lack sufficient transcriptomic support or exhibit inconsistent evidence, and discards features with low quality or confidence levels [58].

The RmCVSA assembly was also annotated using the BRAKER3 pipeline with the Arthropoda protein database [46] as well as transcriptomic data. Available SR sialo transcriptomic data for *R. microplus* were retrieved from GenBank (SRR 3951579-86; SRR10740626-31) [59, 60] using the SRA Toolkit [61]. The single-end and paired-end short reads were used as input in BRAKER3 with the protein database to generate the gene set.

Since a reference genome exists for *R. microplus*, we also utilized a reference-based annotation tool, Liftoff, to lift-over annotations from the reference to the RmCVSA genome assembly. The annotation was performed using the RmJia annotation as the reference genome, where the gene features were mapped to the target assembly, maximizing sequence identity while preserving the structural integrity of each exon, transcript, and gene [62].

The final gene sets for RaCVSA and RmCVSA were functionally annotated using EggNOG mapper [63–65] and InterProScan [66]. Proteins corresponding to the final gene models were annotated with EggNOG mapper against the eggnog database v.6.0 [64] using Diamond [54]. EggNOG maps input protein sequences to orthologous groups (OGs) and provides functional annotations based on precomputed evolutionary relationships and functional annotations from the EggNOG databases [63]. Additionally, InterProScan was used to annotate these proteins against the InterPro database, utilizing HMMER and BLAST for protein domain identification and annotation [66, 67]. To integrate the structural and functional annotations, funannotate [68] was used to combine the BRAKER3 structural annotation with the results from both EggNOG mapper and InterProScan.

The quality of the annotations was estimated using BUSCO in protein mode, which calculates the presence/absence of single-copy marker genes in a genome or an annotated protein set [69, 70].

## Electronic supplementary material

Below is the link to the electronic supplementary material.

Supplementary Material 1

Supplementary Material 2

Supplementary Material 3

Supplementary Material 4

Supplementary Material 5

Supplementary Material 6

Supplementary Material 7

## Data Availability

The genome assembly reference data generated during this study are included within this article and are available in GenBank under project numbers PRJNA819877 (R. microplus nuclear genome and annotation); PRJNA820528 (R. appendiculatus nuclear genome and annotation); OR545517 (R. microplus mitochondrial genome); OR545516 (R. appendiculatus mitochondrial genome); CP094229 (R. microplus CLE); CP094378 (R. appendiculatus CLE).
