# Supplementary figures and images for: Tick genomics through a Nanopore: a low-cost approach for tick genomics
Source: BMC Genomics. 2025 Jul 1;26:591. doi: 10.1186/s12864-025-11733-4 (PMC12211944; doi:10.1186/s12864-025-11733-4)

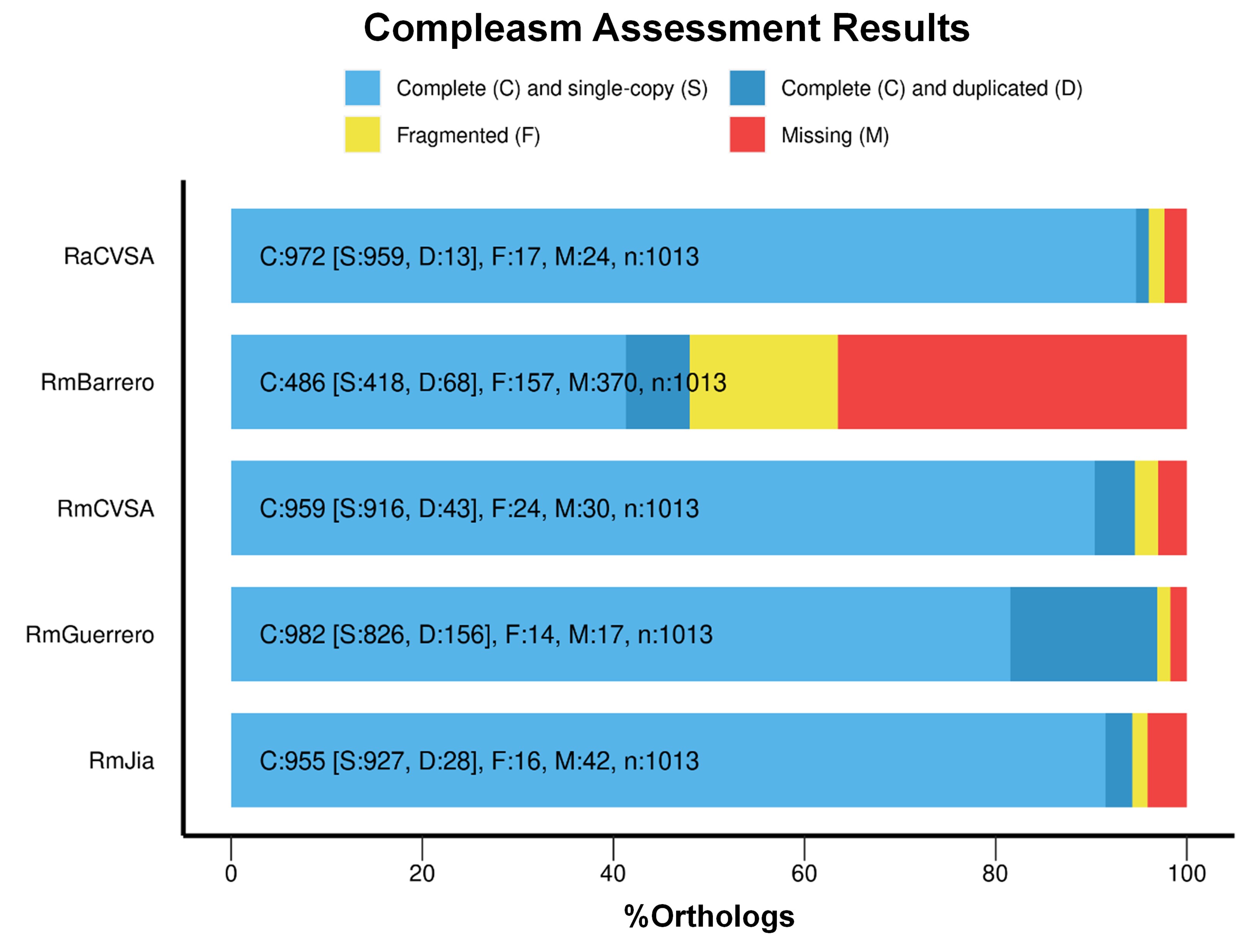

Supplement: Supplementary file 1 — Supplementary Material 1 [file 12864_2025_11733_MOESM1_ESM.jpg]

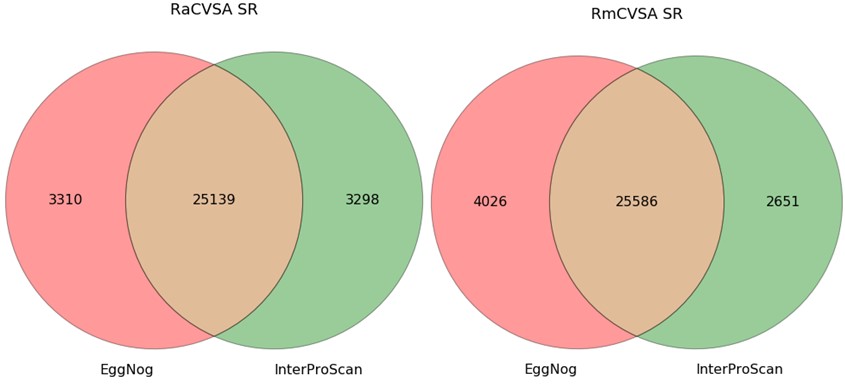

Supplement: Supplementary file 2 — Supplementary Material 2 [file 12864_2025_11733_MOESM2_ESM.jpg]

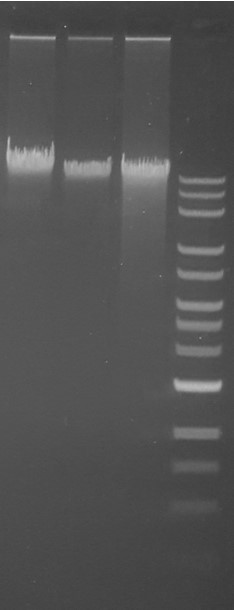

Supplement: Supplementary file 3 — Supplementary Material 3 [file 12864_2025_11733_MOESM3_ESM.jpg]
