# Supplementary material for: Tick genomics through a Nanopore: a low-cost approach for tick genomics
Source: BMC Genomics. 2025 Jul 1;26:591. doi: 10.1186/s12864-025-11733-4 (PMC12211944; doi:10.1186/s12864-025-11733-4)
Supplement: Supplementary file 4 — Supplementary Material 4 [file 12864_2025_11733_MOESM4_ESM.docx]

***Tick genomics through a Nanopore: A low-cost approach for tick genomics***

Christina Meiring^1,2^, Monique Eygelaar^1,2^, Josephus Fourie^3^, Michel Labuschagne^1,2*^

^1^ Clinglobal, B03/04, The Tamarin Commercial Hub, Jacaranda Avenue, Tamarin, 90903, Mauritius

^2^ Clinomics, Uitzich Road, Bainsvlei, Bloemfontein, 9338, South Africa

^3^ Clinvet International Pty (Ltd), 1479 Talmadge Hill South, Waverly, New York 14892, United States of America

^*^Corresponding author

Michel Labuschagne: [michel.labuschagne@clinglobal.com](mailto:michel.labuschagne@clinglobal.com)

Monique Eygelaar: [eygelaarmonique@gmail.com](mailto:eygelaarmonique@gmail.com)

Christina Meiring: [tina.meiring@clinglobal.com](mailto:tina.meiring@clinglobal.com)

Josephus Fourie: [josephus.fourie@clinvet.com](mailto:josephus.fourie@clinvet.com)

Supplementary information

**Supplementary Table 1.** Summary of assembly information for each tick species

|  |  |  | Input | | | | Output | | | | |
| --- | --- | --- | --- | --- | --- | --- | --- | --- | --- | --- | --- |
| Sample | Base calling | Min Read Length | Reads | Bases | Avg Read Len | N50 | Assembler | Assembled Bases | N50 | Contigs | Longest Contig |
| RmCVSA | HAC | 1,000 | 11,883,128 | 128,409,120,366 | 10,806 | 14,747 | Shasta | 3,698,401,431 | 625,896 | 55,920 | 13,803,684 |
| RmCVSA Polished | HAC | 1,000 |  |  |  |  |  | 3,685,168,943 | 648,816 | 42,426 | 13,960,137 |
| RmCVSA PurgeHaplotigs | HAC | 1,000 |  |  |  |  |  | 2,594,159,384 | 1,507,730 | 11,566 | 13,960,137 |
| RmCVSA | HAC | 10,000 | 5,538,025 | 96,910,644,375 | 17,499 | 17,309 |  | 3,776,315,463 | 302,925 | 58,528 | 17,747,350 |
| RmCVSA Polished | HAC | 10,000 |  |  |  |  |  | 3,764,120,979 | 311,143 | 45,881 | 17,807,522 |
| RmCVSA PurgeHaplotigs | HAC | 10,000 |  |  |  |  |  | 2,560,315,105 | 847,825 | 14,407 | 17,807,522 |
| **RmCVSA QuickMerge (FINAL)** | **HAC** |  |  |  |  |  |  | **2,670,281,449** | **1,889,595** | **11,359** | **20,911,290** |
| RmCVSA | HAC |  |  |  |  |  | Flye | 3,777,968,727 | 162,282 | 68,172 | 6,974,318 |
| RmCVSA Polished | HAC |  |  |  |  |  |  | 3,708,801,151 | 169,567 | 65,996 | 6,987,564 |
| RmCVSA PurgeHaplotigs | HAC |  |  |  |  |  |  | 2,834,021,523 | 240,313 | 32,445 | 6,987,564 |
| RmCVSA | SUP | 1,000 | 11,542,891 | 121,969,415,637 | 10,566 | 14,796 | Shasta | 3,807,791,302 | 543,121 | 55,836 | 11,768,953 |
|  |  |  |  |  |  |  |  |  |  |  |  |
|  |  |  |  |  |  |  |  |  |  |  |  |
|  |  |  |  |  |  |  |  |  |  |  |  |
| RmCVSA Polished | SUP | 1,000 |  |  |  |  |  | 3,797,888,284 | 561,016 | 44,091 | 11,854,169 |
| RmCVSA PurgeHaplotigs | SUP | 1,000 |  |  |  |  |  | 2,631,268,645 | 1,411,779 | 11,737 | 11,854,169 |
| RmCVSA | SUP | 10,000 | 5,144,883 | 91,267,205,830 | 17,739 | 17,544 |  | 3,723,257,499 | 320,794 | 57,921 | 9,667,285 |
| RmCVSA Polished | SUP | 10,000 |  |  |  |  |  | 3,709,144,554 | 332,986 | 44,197 | 9,709,536 |
| RmCVSA PurgeHaplotigs | SUP | 10,000 |  |  |  |  |  | 2,603,596,371 | 843,368 | 14,291 | 9,709,536 |
| RmCVSA | SUP |  |  |  |  |  | Flye | 3,710,720,923 | 167,216 | 66,196 | 4,341,594 |
| RmCVSA Polished | SUP |  |  |  |  |  |  | 3,648,853,830 | 174,48 | 64,341 | 4,346,827 |
| RmCVSA PurgeHaplotigs | SUP |  |  |  |  |  |  | 2,837,153,388 | 241,429 | 32,542 | 4,346,827 |
| **RmCVSA QuickMerge (FINAL)** | **SUP** |  |  |  |  |  | Shasta | **2,678,591,779** | **1,707,119** | **11,544** | **26,072,894** |
| RaCVSA | HAC | 1,000 | 18,820,735 | 141,393,533,849 | 7,512 | 10,123 |  | 2,724,122,839 | 119,994 | 97,188 | 10,026,719 |
| RaCVSA Polished | HAC | 1,000 |  |  |  |  |  | 2,709,802,153 | 123,593 | 66,637 | 10,171,704 |
| RaCVSA PurgeHaplotigs | HAC | 1,000 |  |  |  |  |  | 2,302,137,031 | 219,535 | 36,683 | 10,171,704 |
| RaCVSA | HAC | 10,000 | 4,745,853 | 71,928,178,777 | 15,156 | 14,455 |  | 2,314,290,558 | 309,257 | 63,862 | 8,932,820 |
| RaCVSA Polished | HAC | 10,000 |  |  |  |  |  | 2,308,899,760 | 326,907 | 42,357 | 8,993,663 |
| RaCVSA PurgeHaplotigs | HAC | 10,000 |  |  |  |  |  | 2,042,711,349 | 468,267 | 22,756 | 8,993,663 |
| **RaCVSA QuickMerge (FINAL)** | **HAC** |  |  |  |  |  |  | **2,366,026,835** | **679,575** | **33,490** | **20,760,809** |
| RaCVSA | SUP | 1,000 | 19,951,829 | 148,376,224,876 | 7,436 | 9,943 |  | 3,058,307,341 | 72,898 | 120,747 | 4,814,177 |
| RaCVSA Polished | SUP | 1,000 |  |  |  |  |  | 3,047,657,588 | 75,406 | 89,092 | 4,858,747 |
| RaCVSA PurgeHaplotigs | SUP | 1,000 |  |  |  |  |  | 2,449,999,517 | 113,938 | 45,385 | 4,858,747 |
| RaCVSA | SUP | 10,000 | 5,015,237 | 73,576,387,148 | 14,670 | 14,114 |  | 2,351,928,790 | 233,254 | 67,205 | 6,862,136 |
| RaCVSA Polished | SUP | 10,000 |  |  |  |  |  | 2,351,508,852 | 246,459 | 44,725 | 6,914,502 |
| RaCVSA PurgeHaplotigs | SUP | 10,000 |  |  |  |  |  | 2,078,974,711 | 376,163 | 25,013 | 6,914,502 |
| **RaCVSA QuickMerge (FINAL)** | **SUP** |  |  |  |  |  |  | **2,545,458,806** | **686,602** | **23,030** | **19,469,694** |


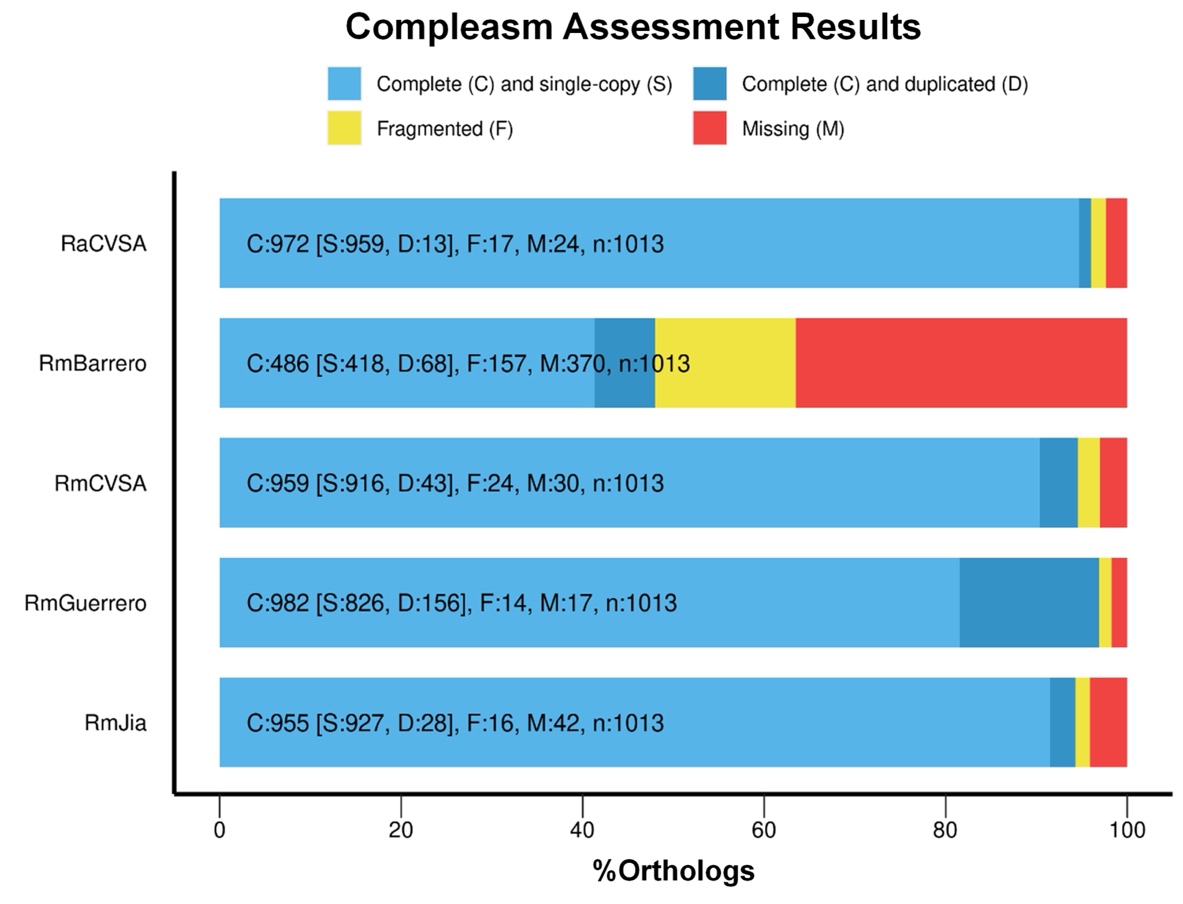


**Supplementary Figure 1.** Compleasm results for RaCVSA and RmCVSA genomes generated during this study as well as the *R. microplus* genomes available in GenBank.

**Supplementary Table 2.** Summary of annotation information for RaCVSA and RmCVSA.

| Genome and annotation input | RaCVSA SR | RmCVSA SR | RmJia | RmCVSA (Liftoff) |
| --- | --- | --- | --- | --- |
|  | Short-reads and OrthoDB proteins | Short-reads and OrthoDB proteins | NA | NA |
| Total predicted genes | 52,412 | 60,935 | 29,870 | 25,307 |
| Genes supported by external evidence | 25,400 | 30,501 | NA | NA |
| Functional Annotations | 31,747 | 32,263 | 29,866 | 25,307 |
| Total genes in GenBank annotated assembly | 52,408 | 59,923 | NA | NA |
| Total protein coding genes in GenBank annotated assembly | 52,404 | 59,922 | NA | NA |

SR: short-read


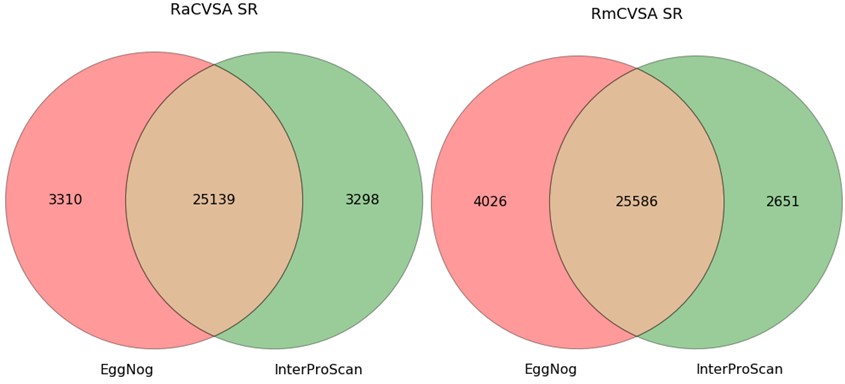


**Supplementary Figure 2.** Venn diagram comparisons of the functional annotations identified by EggNog and InterProScan.

**Supplementary Table 3.** Bioinformatic software tools and algorithms used in this study.

| **Software** | **Version** | **Source/reference** |
| --- | --- | --- |
| AUGUSTUS | 3.5.0 | [Gaius-Augustus/Augustus: Genome annotation with AUGUSTUS (github.com)](https://github.com/Gaius-Augustus/Augustus) |
| BEDtools | 2.31.1 | <https://bedtools.readthedocs.io/en/latest/> |
| BLAST | 2.12.0 | <https://blast.ncbi.nlm.nih.gov/Blast.cgi?PAGE_TYPE=BlastDocs&DOC_TYPE=Download> |
| BRAKER | 3.0.7 | [Gaius-Augustus/BRAKER: BRAKER is a pipeline for fully automated prediction of protein coding gene structures with GeneMark-ES/ET/EP/ETP and AUGUSTUS in novel eukaryotic genomes (github.com)](https://github.com/Gaius-Augustus/BRAKER) |
| BUSCO | 5.6.1 | <https://gitlab.com/ezlab/busco> |
| Compleasm | 0.2.5 | [huangnengCSU/compleasm: A genome completeness evaluation tool based on miniprot (github.com)](https://github.com/huangnengCSU/compleasm) |
| DIAMOND | 2.1.9 | [bbuchfink/diamond: Accelerated BLAST compatible local sequence aligner. (github.com)](https://github.com/bbuchfink/diamond) |
| EggNOG-mapper | 2.1.12 | [eggnogdb/eggnog-mapper: Fast genome-wide functional annotation through orthology assignment (github.com)](https://github.com/eggnogdb/eggnog-mapper) |
| Flye | 2.9 | https://github.com/fenderglass/Flye |
| Funannotate | 1.8.17 | [nextgenusfs/funannotate: Eukaryotic Genome Annotation Pipeline (github.com)](https://github.com/nextgenusfs/funannotate) |
| GenSAS | 6 | <https://www.gensas.org/> |
| GeneMark |  | http://exon.gatech.edu/GeneMark/ |
| Guppy | 4.0.14 & 5.0.11 | <https://community.nanoporetech.com/downloads/guppy> |
| HiSat2 | 2.2.1 | [DaehwanKimLab/hisat2: Graph-based alignment (Hierarchical Graph FM index) (github.com)](https://github.com/DaehwanKimLab/hisat2) |
| InterProScan | 5.66-98.0 | [ebi-pf-team/interproscan: Genome-scale protein function classification (github.com)](https://github.com/ebi-pf-team/interproscan) |
| Liftoff | 1.6.3 | [agshumate/Liftoff: An accurate GFF3/GTF lift over pipeline (github.com)](https://github.com/agshumate/Liftoff) |
| MAFFT | 7.45 | [MAFFT - a multiple sequence alignment program (cbrc.jp)](https://mafft.cbrc.jp/alignment/software/) |
| MarginPolish | 1.3.0 | <https://github.com/UCSC-nanopore-cgl/MarginPolish> |
| Medaka |  | <https://github.com/nanoporetech/medaka> |
| Minimap2 | 2.17 | <https://github.com/lh3/minimap2> |
| MITOS |  | http://mitos.bioinf.uni-leipzig.de |
| MRBAYES | 3.2.6 | (Huelsenbeck and Ronquist, 2001) |
| MUMmer | 4 | (Marçais et al., 2018) |
| NanoFilt | 1.6.0 | https://github.com/wdecoster/nanofilt |
| NanoStat | 2.8.0 | https://github.com/wdecoster/nanostat |
| ProtHint |  | [gatech-genemark/ProtHint: Protein hint generation pipeline for gene finding in eukaryotic genomes (github.com)](https://github.com/gatech-genemark/ProtHint) |
| PurgeHaplotigs |  | <https://github.com/skingan/purge_haplotigs_multiBAM> |
| QuickMerge | 0.3 | [mahulchak/quickmerge: A simple and fast metassembler and assembly gap filler designed for long molecule based assemblies. (github.com)](https://github.com/mahulchak/quickmerge) |
| RAST | 2 | [RAST Server - RAST Annotation Server (nmpdr.org)](https://rast.nmpdr.org/) |
| RepeatModeler | 2.0.1 | [Available Tools \| GenSAS v6.0](https://www.gensas.org/tools) |
| RepeatModeler2 | 2.0.5 | [photocyte/repeatModeler2_nf: Nextflow workflow to easily run RepeatModeler with conda and singularity (github.com)](https://github.com/photocyte/repeatModeler2_nf) |
| RepeatMasker | 4.1.6 | [rmhubley/RepeatMasker: RepeatMasker is a program that screens DNA sequences for interspersed repeats and low complexity DNA sequences. (github.com)](https://github.com/rmhubley/RepeatMasker) |
| SAMtools | 1.9 | <https://github.com/samtools/samtools> |
| Seqtk | 1.3 | <https://github.com/lh3/seqtk> |
| Shasta | 0.7.0 | <https://github.com/chanzuckerberg/shasta> |
| Spaln2 |  | [ogotoh/spaln: Genome mapping and spliced alignment of cDNA or amino acid sequences (github.com)](https://github.com/ogotoh/spaln) |
| StringTie2 | 2.2.1 | [skovaka/stringtie2: Transcript assembly and quantification for RNA-Seq (github.com)](https://github.com/skovaka/stringtie2) |
| TSEBRA | 1.1.2.3 | https://github.com/Gaius-Augustus/TSEBRA |
