# Supplementary material for: Tick genomics through a Nanopore: a low-cost approach for tick genomics
Source: BMC Genomics. 2025 Jul 1;26:591. doi: 10.1186/s12864-025-11733-4 (PMC12211944; doi:10.1186/s12864-025-11733-4)
Supplement: Supplementary file 5 — Supplementary Material 5 [file 12864_2025_11733_MOESM5_ESM.docx]

Supplementary Table 1

|  |  |  | Input | | | | Output | | | | |
| --- | --- | --- | --- | --- | --- | --- | --- | --- | --- | --- | --- |
| Sample | Base calling | Min Read Length | Reads | Bases | Avg Read Len | N50 | Assembler | Assembled Bases | N50 | Contigs | Longest Contig |
| RmCVSA | HAC | 1,000 | 11,883,128 | 128,409,120,366 | 10,806 | 14,747 | Shasta | 3,698,401,431 | 625,896 | 55,920 | 13,803,684 |
| RmCVSA Polished | HAC | 1,000 |  |  |  |  |  | 3,685,168,943 | 648,816 | 42,426 | 13,960,137 |
| RmCVSA PurgeHaplotigs | HAC | 1,000 |  |  |  |  |  | 2,594,159,384 | 1,507,730 | 11,566 | 13,960,137 |
| RmCVSA | HAC | 10,000 | 5,538,025 | 96,910,644,375 | 17,499 | 17,309 |  | 3,776,315,463 | 302,925 | 58,528 | 17,747,350 |
| RmCVSA Polished | HAC | 10,000 |  |  |  |  |  | 3,764,120,979 | 311,143 | 45,881 | 17,807,522 |
| RmCVSA PurgeHaplotigs | HAC | 10,000 |  |  |  |  |  | 2,560,315,105 | 847,825 | 14,407 | 17,807,522 |
| **RmCVSA QuickMerge (FINAL)** | **HAC** |  |  |  |  |  |  | **2,670,281,449** | **1,889,595** | **11,359** | **20,911,290** |
| RmCVSA | HAC |  |  |  |  |  | Flye | 3,777,968,727 | 162,282 | 68,172 | 6,974,318 |
| RmCVSA Polished | HAC |  |  |  |  |  |  | 3,708,801,151 | 169,567 | 65,996 | 6,987,564 |
| RmCVSA PurgeHaplotigs | HAC |  |  |  |  |  |  | 2,834,021,523 | 240,313 | 32,445 | 6,987,564 |
| RmCVSA | SUP | 1,000 | 11,542,891 | 121,969,415,637 | 10,566 | 14,796 | Shasta | 3,807,791,302 | 543,121 | 55,836 | 11,768,953 |
|  |  |  |  |  |  |  |  |  |  |  |  |
|  |  |  |  |  |  |  |  |  |  |  |  |
|  |  |  |  |  |  |  |  |  |  |  |  |
| RmCVSA Polished | SUP | 1,000 |  |  |  |  |  | 3,797,888,284 | 561,016 | 44,091 | 11,854,169 |
| RmCVSA PurgeHaplotigs | SUP | 1,000 |  |  |  |  |  | 2,631,268,645 | 1,411,779 | 11,737 | 11,854,169 |
| RmCVSA | SUP | 10,000 | 5,144,883 | 91,267,205,830 | 17,739 | 17,544 |  | 3,723,257,499 | 320,794 | 57,921 | 9,667,285 |
| RmCVSA Polished | SUP | 10,000 |  |  |  |  |  | 3,709,144,554 | 332,986 | 44,197 | 9,709,536 |
| RmCVSA PurgeHaplotigs | SUP | 10,000 |  |  |  |  |  | 2,603,596,371 | 843,368 | 14,291 | 9,709,536 |
| RmCVSA | SUP |  |  |  |  |  | Flye | 3,710,720,923 | 167,216 | 66,196 | 4,341,594 |
| RmCVSA Polished | SUP |  |  |  |  |  |  | 3,648,853,830 | 174,48 | 64,341 | 4,346,827 |
| RmCVSA PurgeHaplotigs | SUP |  |  |  |  |  |  | 2,837,153,388 | 241,429 | 32,542 | 4,346,827 |
| **RmCVSA QuickMerge (FINAL)** | **SUP** |  |  |  |  |  | Shasta | **2,678,591,779** | **1,707,119** | **11,544** | **26,072,894** |
| RaCVSA | HAC | 1,000 | 18,820,735 | 141,393,533,849 | 7,512 | 10,123 |  | 2,724,122,839 | 119,994 | 97,188 | 10,026,719 |
| RaCVSA Polished | HAC | 1,000 |  |  |  |  |  | 2,709,802,153 | 123,593 | 66,637 | 10,171,704 |
| RaCVSA PurgeHaplotigs | HAC | 1,000 |  |  |  |  |  | 2,302,137,031 | 219,535 | 36,683 | 10,171,704 |
| RaCVSA | HAC | 10,000 | 4,745,853 | 71,928,178,777 | 15,156 | 14,455 |  | 2,314,290,558 | 309,257 | 63,862 | 8,932,820 |
| RaCVSA Polished | HAC | 10,000 |  |  |  |  |  | 2,308,899,760 | 326,907 | 42,357 | 8,993,663 |
| RaCVSA PurgeHaplotigs | HAC | 10,000 |  |  |  |  |  | 2,042,711,349 | 468,267 | 22,756 | 8,993,663 |
| **RaCVSA QuickMerge (FINAL)** | **HAC** |  |  |  |  |  |  | **2,366,026,835** | **679,575** | **33,490** | **20,760,809** |
| RaCVSA | SUP | 1,000 | 19,951,829 | 148,376,224,876 | 7,436 | 9,943 |  | 3,058,307,341 | 72,898 | 120,747 | 4,814,177 |
| RaCVSA Polished | SUP | 1,000 |  |  |  |  |  | 3,047,657,588 | 75,406 | 89,092 | 4,858,747 |
| RaCVSA PurgeHaplotigs | SUP | 1,000 |  |  |  |  |  | 2,449,999,517 | 113,938 | 45,385 | 4,858,747 |
| RaCVSA | SUP | 10,000 | 5,015,237 | 73,576,387,148 | 14,670 | 14,114 |  | 2,351,928,790 | 233,254 | 67,205 | 6,862,136 |
| RaCVSA Polished | SUP | 10,000 |  |  |  |  |  | 2,351,508,852 | 246,459 | 44,725 | 6,914,502 |
| RaCVSA PurgeHaplotigs | SUP | 10,000 |  |  |  |  |  | 2,078,974,711 | 376,163 | 25,013 | 6,914,502 |
| **RaCVSA QuickMerge (FINAL)** | **SUP** |  |  |  |  |  |  | **2,545,458,806** | **686,602** | **23,030** | **19,469,694** |
