# Supplementary material for: Tick genomics through a Nanopore: a low-cost approach for tick genomics
Source: BMC Genomics. 2025 Jul 1;26:591. doi: 10.1186/s12864-025-11733-4 (PMC12211944; doi:10.1186/s12864-025-11733-4)
Supplement: Supplementary file 6 — Supplementary Material 6 [file 12864_2025_11733_MOESM6_ESM.docx]

| Genome and annotation input | RaCVSA SR | RmCVSA SR | RmJia | RmCVSA (Liftoff) |
| --- | --- | --- | --- | --- |
|  | Short-reads and OrthoDB proteins | Short-reads and OrthoDB proteins | NA | NA |
| Total predicted genes | 52,412 | 60,935 | 29,870 | 25,307 |
| Genes supported by external evidence | 25,400 | 30,501 | NA | NA |
| Functional Annotations | 31,747 | 32,263 | 29,866 | 25,307 |
| Total genes in GenBank annotated assembly | 52,408 | 59,923 | NA | NA |
| Total protein coding genes in GenBank annotated assembly | 52,404 | 59,922 | NA | NA |

SR: short-read
