# Supplementary material for: Tick genomics through a Nanopore: a low-cost approach for tick genomics
Source: BMC Genomics. 2025 Jul 1;26:591. doi: 10.1186/s12864-025-11733-4 (PMC12211944; doi:10.1186/s12864-025-11733-4)
Supplement: Supplementary file 7 — Supplementary Material 7 [file 12864_2025_11733_MOESM7_ESM.docx]

| **Software** | **Version** | **Source/reference** |
| --- | --- | --- |
| AUGUSTUS | 3.5.0 | [Gaius-Augustus/Augustus: Genome annotation with AUGUSTUS (github.com)](https://github.com/Gaius-Augustus/Augustus) |
| BEDtools | 2.31.1 | <https://bedtools.readthedocs.io/en/latest/> |
| BLAST | 2.12.0 | <https://blast.ncbi.nlm.nih.gov/Blast.cgi?PAGE_TYPE=BlastDocs&DOC_TYPE=Download> |
| BRAKER | 3.0.7 | [Gaius-Augustus/BRAKER: BRAKER is a pipeline for fully automated prediction of protein coding gene structures with GeneMark-ES/ET/EP/ETP and AUGUSTUS in novel eukaryotic genomes (github.com)](https://github.com/Gaius-Augustus/BRAKER) |
| BUSCO | 5.6.1 | <https://gitlab.com/ezlab/busco> |
| Compleasm | 0.2.5 | [huangnengCSU/compleasm: A genome completeness evaluation tool based on miniprot (github.com)](https://github.com/huangnengCSU/compleasm) |
| DIAMOND | 2.1.9 | [bbuchfink/diamond: Accelerated BLAST compatible local sequence aligner. (github.com)](https://github.com/bbuchfink/diamond) |
| EggNOG-mapper | 2.1.12 | [eggnogdb/eggnog-mapper: Fast genome-wide functional annotation through orthology assignment (github.com)](https://github.com/eggnogdb/eggnog-mapper) |
| Flye | 2.9 | https://github.com/fenderglass/Flye |
| Funannotate | 1.8.17 | [nextgenusfs/funannotate: Eukaryotic Genome Annotation Pipeline (github.com)](https://github.com/nextgenusfs/funannotate) |
| GenSAS | 6 | <https://www.gensas.org/> |
| GeneMark |  | http://exon.gatech.edu/GeneMark/ |
| Guppy | 4.0.14 & 5.0.11 | <https://community.nanoporetech.com/downloads/guppy> |
| HiSat2 | 2.2.1 | [DaehwanKimLab/hisat2: Graph-based alignment (Hierarchical Graph FM index) (github.com)](https://github.com/DaehwanKimLab/hisat2) |
| InterProScan | 5.66-98.0 | [ebi-pf-team/interproscan: Genome-scale protein function classification (github.com)](https://github.com/ebi-pf-team/interproscan) |
| Liftoff | 1.6.3 | [agshumate/Liftoff: An accurate GFF3/GTF lift over pipeline (github.com)](https://github.com/agshumate/Liftoff) |
| MAFFT | 7.45 | [MAFFT - a multiple sequence alignment program (cbrc.jp)](https://mafft.cbrc.jp/alignment/software/) |
| MarginPolish | 1.3.0 | <https://github.com/UCSC-nanopore-cgl/MarginPolish> |
| Medaka |  | <https://github.com/nanoporetech/medaka> |
| Minimap2 | 2.17 | <https://github.com/lh3/minimap2> |
| MITOS |  | http://mitos.bioinf.uni-leipzig.de |
| MRBAYES | 3.2.6 | (Huelsenbeck and Ronquist, 2001) |
| MUMmer | 4 | (Marçais et al., 2018) |
| NanoFilt | 1.6.0 | https://github.com/wdecoster/nanofilt |
| NanoStat | 2.8.0 | https://github.com/wdecoster/nanostat |
| ProtHint |  | [gatech-genemark/ProtHint: Protein hint generation pipeline for gene finding in eukaryotic genomes (github.com)](https://github.com/gatech-genemark/ProtHint) |
| PurgeHaplotigs |  | <https://github.com/skingan/purge_haplotigs_multiBAM> |
| QuickMerge | 0.3 | [mahulchak/quickmerge: A simple and fast metassembler and assembly gap filler designed for long molecule based assemblies. (github.com)](https://github.com/mahulchak/quickmerge) |
| RAST | 2 | [RAST Server - RAST Annotation Server (nmpdr.org)](https://rast.nmpdr.org/) |
| RepeatModeler | 2.0.1 | [Available Tools \| GenSAS v6.0](https://www.gensas.org/tools) |
| RepeatModeler2 | 2.0.5 | [photocyte/repeatModeler2_nf: Nextflow workflow to easily run RepeatModeler with conda and singularity (github.com)](https://github.com/photocyte/repeatModeler2_nf) |
| RepeatMasker | 4.1.6 | [rmhubley/RepeatMasker: RepeatMasker is a program that screens DNA sequences for interspersed repeats and low complexity DNA sequences. (github.com)](https://github.com/rmhubley/RepeatMasker) |
| SAMtools | 1.9 | <https://github.com/samtools/samtools> |
| Seqtk | 1.3 | <https://github.com/lh3/seqtk> |
| Shasta | 0.7.0 | <https://github.com/chanzuckerberg/shasta> |
| Spaln2 |  | [ogotoh/spaln: Genome mapping and spliced alignment of cDNA or amino acid sequences (github.com)](https://github.com/ogotoh/spaln) |
| StringTie2 | 2.2.1 | [skovaka/stringtie2: Transcript assembly and quantification for RNA-Seq (github.com)](https://github.com/skovaka/stringtie2) |
| TSEBRA | 1.1.2.3 | https://github.com/Gaius-Augustus/TSEBRA |
